# Genome-Wide Association Mapping for Kernel and Malting Quality Traits Using Historical European Barley Records

**DOI:** 10.1371/journal.pone.0110046

**Published:** 2014-11-05

**Authors:** Inge E. Matthies, Marcos Malosetti, Marion S. Röder, Fred van Eeuwijk

**Affiliations:** 1 Department of Gene and genome mapping, Leibniz Institute of Plant Genetics and Crop Plant Research (IPK), Gatersleben, Sachsen-Anhalt, Germany; 2 Biometris, Wageningen University and Research Centre, Wageningen, Gelderland, The Netherlands; Kansas State University, United States of America

## Abstract

Malting quality is an important trait in breeding barley (*Hordeum vulgare* L.). It requires elaborate, expensive phenotyping, which involves micro-malting experiments. Although there is abundant historical information available for different cultivars in different years and trials, that historical information is not often used in genetic analyses. This study aimed to exploit historical records to assist in identifying genomic regions that affect malting and kernel quality traits in barley. This genome-wide association study utilized information on grain yield and 18 quality traits accumulated over 25 years on 174 European spring and winter barley cultivars combined with diversity array technology markers. Marker-trait associations were tested with a mixed linear model. This model took into account the genetic relatedness between cultivars based on principal components scores obtained from marker information. We detected 140 marker-trait associations. Some of these associations confirmed previously known quantitative trait loci for malting quality (on chromosomes 1H, 2H, and 5H). Other associations were reported for the first time in this study. The genetic correlations between traits are discussed in relation to the chromosomal regions associated with the different traits. This approach is expected to be particularly useful when designing strategies for multiple trait improvements.

## Introduction

Barley (*Hordeum vulgare* L.) is a major cereal crop in Europe. It ranks fourth in worldwide production, after wheat, rice, and maize. It is grown for feed, food, and malting. Most of the malt produced is used for brewing beer and, to a lesser extent, for distilling (e.g., whiskey). In Europe, two-rowed spring cultivars are used mainly for malting and brewing; six-rowed winter barleys are predominantly used for food. However, six-rowed barley has been increasingly used for malting in Europe, following the trend started in the US. Therefore, depending on the end-use, there are two primary aims in breeding barley: 1) superior food and feed quality with high protein content, and 2) high malting quality with high starch and low protein contents. Improving the malting quality is a central goal in breeding, in addition to improving the yield of barley. Malting quality is a complex trait, because it consists of several components, and all are polygenic. Moreover, the definition of high malting quality is not straightforward; it depends on the processing and brewing methods. In general, the main breeding goals for malting barley are high malting extract, low protein content, good solubility properties, good kernel formation, and low glume content.

For the past 80 years, to optimize the malting traits in barley, breeders mainly focused on a narrow gene pool of spring barley types [1]; the most important quality parameters to optimize were the amounts of soluble protein, extract, raw protein, and friability. Further improvements in malting quality must rely on new combinations of genes and germplasms. Molecular marker-assisted selection (MAS) schemes have been applied to developing barley varieties with improved malting quality traits. Those studies have identified many quantitative trait loci (QTL) in barley [2]–[4]. MAS strategies have facilitated gene pyramiding techniques to acquire advantageous alleles from different loci. With MAS, the breeding efficiency can be improved by eliminating undesired genotypes at early stages, which can reduce time and costs [4]–[7]. The genome-wide association approach provides a good basis for selection strategies in any breeding program.

The identification of barley genomic regions that influence yield and malting properties will increase our understanding of the genetics and promote the development of cultivars with improved kernel and malting quality. The genetic and biochemical bases of malting quality in barley have been addressed previously [2], [8], [9]. However, quantification of malting quality parameters requires elaborate, expensive phenotypic analyses.

Typically, the high cost of assessing malting quality in barley lines is due to expensive equipment, laboratory facilities, and experienced personnel. Moreover, assessing malting and brewing quality requires substantial amounts of grain (100–1,000 g), which is often not feasible in the early generations of a breeding cycle. In addition, some malting quality parameters can only be determined in time-consuming, wet lab analyses. These limitations may be overcome with the use of historical phenotypic data recorded in statistical year books, like those from the Deutsche Braugerstengemeinschaft or the European Brewery Association. These resources may provide a cost-effective approach. The complex dataset considered in the present study may serve as a valid resource for breeding barley varieties with high malting quality.

In addition, the identification of marker-trait associations (MTAs) may represent a cost effective strategy for selecting traits that are typically expensive to identify in MAS schemes [2], [3], [10]. Molecular markers and QTLs have been described for numerous traits in barley, and major genes have been detected in segregating populations derived from biparental crosses [3], [10]–[14]. The use of genome-wide association mapping for QTL detection has attracted interest in agricultural settings, due to the recent availability of high-throughput genotyping technology and the development of new statistics methodologies [15]–[17].

Association mapping, also known as linkage disequilibrium (LD) mapping, represents an interesting alternative to traditional linkage analysis. It provides the advantages of (i) wider genomic diversity than provided by biparental segregating populations, (ii) high mapping resolution, by exploiting historical recombinations in the population, and (iii) rapid results, because it is not necessary to create a segregating population [18], [19]. In combination with high-density genotyping, association mapping can resolve complex trait variation down to the sequence level by incorporating historical recombination events that occurred at the population level [20], [21].

Two association mapping methodologies are widely used in plants. The first is a candidate gene approach, which relates polymorphisms in candidate genes to phenotypic variations in traits. The second approach is a genome-wide association study (GWAS), which relates polymorphisms of anonymous markers to trait variations [16], [22]. Candidate gene studies are widely conducted in crop plant species, including barley and maize. Those studies aim to detect functional markers that directly impact the trait of interest [23]–[27]. The GWAS approach has recently benefitted from the advent of cost-effective high throughput marker technology, like Diversity Array Technology (DArT) [28] and Illumina Bead Chips or Bead arrays [29], [30]. High marker coverage is required for conducting a GWAS, but the potential of this approach has been demonstrated in barley [15], [22], [30]–[35].

DArT markers are bi-allelic, dominant markers. A single DArT assay can genotype thousands of SNPs and insertions/deletions across the genome simultaneously. Barley was one of the first plant species for which DArT markers became available [36]–[38]. The integrated barley consensus map now contains 3,542 markers, including DArT markers. This map has been used to locate meaningful associations [39]. The first examples of applying DArT marker technology to *Hordeum* included a GWAS conducted to detect yield-associated genes [40] and a QTL mapping study conducted to identify net blotch resistance in a segregating population [41]. Other examples included the study of linkage disequilibrium (LD) and population structures in association studies that aimed to identify powdery mildew and yield components in barley [42]–[45]. Another study associated DArT markers with malting quality characteristics from two row Canadian barley lines [46]. In another GWAS, 138 wild barley accessions were genotyped with DArT markers and SNP markers from the Illumina Golden Gate Assay to detect genomic regions associated with spot blotch resistance [47].

An important issue in GWAS is that the population structure which arises from heterogeneous genetic relatedness between entries in the association panel can cause high LDs between unlinked loci [48]. When LDs between markers and traits occur as a consequence of the population structure, they are called false positives or spurious associations. Therefore, a statistical model must account for genetic relatedness, typically by choosing an appropriate mixed linear model that accommodates genetic covariance between observations [44], [49], [50]. A wide range of models have been proposed that account in one way or another for relationships between genotypes [18], [19], [49]–[54]. Population structure is particularly prominent in self-pollinating barley [17], [33]; it causes clear spurious associations between spike morphologies (two- versus six-rowed types) and between growth habits and vernalization requirements (winter and spring genotypes) [22], [55]–[59]. The barley collection used in the present study exhibited a combination of those characteristics. Therefore, proper consideration of population structure was required to assure meaningful MTAs.

This study aimed to identify chromosomal regions that influenced kernel and malting quality parameters in barley, based on a diverse set of cultivars and historical phenotypic data. The approach included (*i*) genotyping the germplasm with DArT markerDArT markers, (*ii*) investigating the degree of intrachromosomal LD decay within this barley collection, and (*iii*) performing a GWAS with a mixed linear model approach.

## Results

### Phenotypic data analysis

Based on the available historical data, we obtained best linear unbiased estimators (BLUEs) for grain yield, eight kernel traits, and ten malting quality traits for each cultivar (Table S1). Inspection of residual plots showed no deviations from model assumptions (Figure S1). Traits expressed in percentages were log-transformed before analysis to stabilize the variance. Summary statistics of the adjusted means are shown in Table 1. A large range of variation was observed for most traits, including soluble nitrogen (solN), grain yield (GY), thousand grain weight (TGW), soluble protein (SolP), and saccharification number (VZ45). In general, broad sense heritabilities were above 0.4, with few exceptions, which indicated that a relatively large genetic component was involved in the determination of the observed trait variation (Table 1).

**Table 1:** Summary statistics of the nine agronomic and 10 malting quality traits considered in this study.

Trait	Units	N	Min	Max	Mean	Std. dev.	h^2^
**Agronomic traits**							
GY	[dt/ha]	103	52.5	83.1	71.0	7.5	0.60
TGW	[g]	174	37.8	59.1	46.2	3.7	0.65
HLW	[kg]	165	65.3	72.8	68.7	1.4	0.47
KF	[1]–[9]	131	2.3	6.4	4.6	0.9	0.78
GF	[1]–[9]	131	1.6	6.7	4.4	1.1	0.78
SF <2.2 mm	[%]	159	0.2	7.5	1.9	1.5	0.39
SF 2.2–2.5 mm	[%]	157	1.0	30.3	8.0	5.9	0.24
SF >2.8 mm	[%]	174	20.0	89.6	60.6	17.0	0.57
K_RP	[%]	168	9.1	13.5	11.2	0.9	0.55
**Malting quality traits**							
M_RP	[%]	126	8.9	11.8	10.1	0.6	0.22
solN	[mg/100 g DM]	126	559.0	817.9	706.4	57.8	0.45
solP	[%]	126	33.9	52.3	44.0	3.9	0.50
Visc	[mPas]	95	1.4	2.4	1.6	0.1	0.79
Col	[EBC]	112	2.7	6.0	3.6	0.4	0.12
Fria	[%]	125	33.9	95.4	79.9	13.0	0.54
VZ45	[%]	125	31.2	49.6	40.7	4.0	0.39
Extr	[%]	125	77.1	85.1	81.7	1.6	0.64
FiAt	[%]	114	77.7	83.8	81.5	1.2	0.21
MQI	–	114	3.4	10.2	7.5	1.4	0.62

N = number of varieties, h^2 = ^broad sense heritability. h^2^ was obtained by fitting genotypes as random terms in the statistical model. Trait abbreviations: GY = grain yield, TGW = thousand grain weight, HLW = hectoliter weight, KF = kernel formation, GF = glume fineness, SF = sieve fraction (less than 2.2 mm, 2.2–2.5 mm, or more than 2.8 mm), K_RP = raw kernel protein content, M_RP = raw malt protein content, solN = soluble nitrogen, solP = soluble protein, Visc = viscosity, Col = color, Fria = friability, VZ45 = saccharification number, Extr = malt extract, FiAt = final attenuation, MQI = Malting quality index.

**Figure 1:**
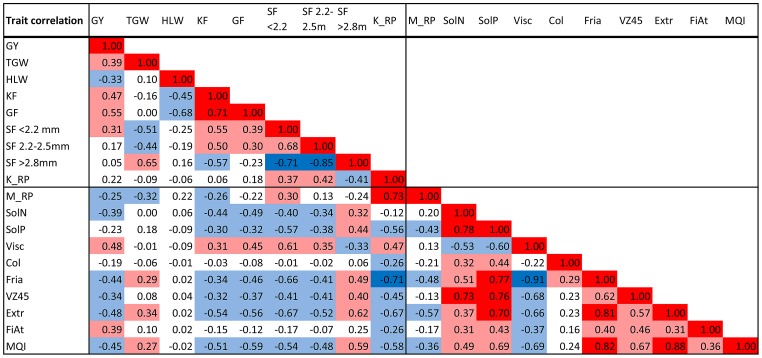
Correlation matrix of yield, kernel quality, and malting quality parameters based on BLUES for each cultivar. The Pearson coefficients of the two sided test are given only for the significant phenotypic trait correlations. BLUES = best linear unbiased estimators, GY = grain yield, MY = marketable yield, TGW = thousand grain weight, HLW = hectoliter weight, KF = kernel formation, GF = glume fineness, SF = sieve fraction, K_RP = raw kernel protein content, M_RP = raw malt protein content, solN = soluble nitrogen, solP = soluble protein, Visc = viscosity, Col = color, Fria = friability, VZ45 = saccharification number VZ45°C, Extr = malt extract, FiAt = final attenuation, MQI = malting quality index.

The correlations among all considered traits are shown in Figure 1. In general, correlations were moderate, with absolute values ranging between 0.30 and 0.70. Strong positive correlations were found between malt extract and the malting quality index (MQI) (r = 0.88), malt extract and friability (r = 0.81); and between MQI and friability (r = 0.82). Furthermore, a high correlation (r = 0.78) was observed between soluble nitrogen (SolN) and soluble protein (SolP). SolN and SolP were also related to color and to the saccharification number (VZ45), as reflected in their relatively high correlations with VZ45 (r = 0.73, and r = 0.76, respectively). Four highly negative correlations were observed between friability and viscosity (r = −0.91); between a larger grain fraction (>2.8 mm) and two smaller grain fractions (r = −0.85 and r = −0.71); and between friability and kernel raw protein (K_RP; r = −0.71). Strongly negative correlations were also observed between malt extract and sieve fractions (SF) <2.2 mm, the K_RP, and viscosity (r = −0.67, −0.67, and r = −0.66, respectively); and between viscosity and VZ45 (r = −0.68). The GY and TGW showed only moderate or low correlations with agronomic and malting quality traits. Both, hectoliter weight (HLW) and color showed low correlations with all other traits. Overall, the correlations among malting quality traits were higher than the correlations among agronomic quality traits. In general, the correlations among agronomic and malting quality traits were moderate to low, which hinted that the genetic determinants of these two types of quality parameters were relatively independent.

### Genotyping with DArT markers

The original set of 1088 DArT markers was reduced to 839, because we discarded monomorphic markers, markers with rare alleles (minor allele frequency <0.05), and markers missing more than 10% of the values. The marker map showed a high genomic coverage, with a density of about one marker in every 5 cM for most genomic regions, except for chromosome 4H, which showed some inter-marker distances larger than 15 cM (Table 2; Figure S2).

**Table 2:** Summary statistics of the 839 DArT markers used in this study.

Chromosome	Length [cM]	No. of markers	Median inter-marker distance	95^th^ percentile of the inter-marker distance
1H	148.1	98	0.2	5.4
2H	163.2	172	0.2	4.9
3H	177.6	138	<0.1	6.1
4H	147.4	57	0.3	16.9
5H	185.3	112	0.5	5.5
6H	141.4	119	<0.1	6.9
7H	160.2	143	<0.1	5.2
**Genome**	**1123.1**	**839**	**0.1**	**5.8**

### Population structure and intrachromosomal linkage disequilibrium (LD)

**Figure 2:**
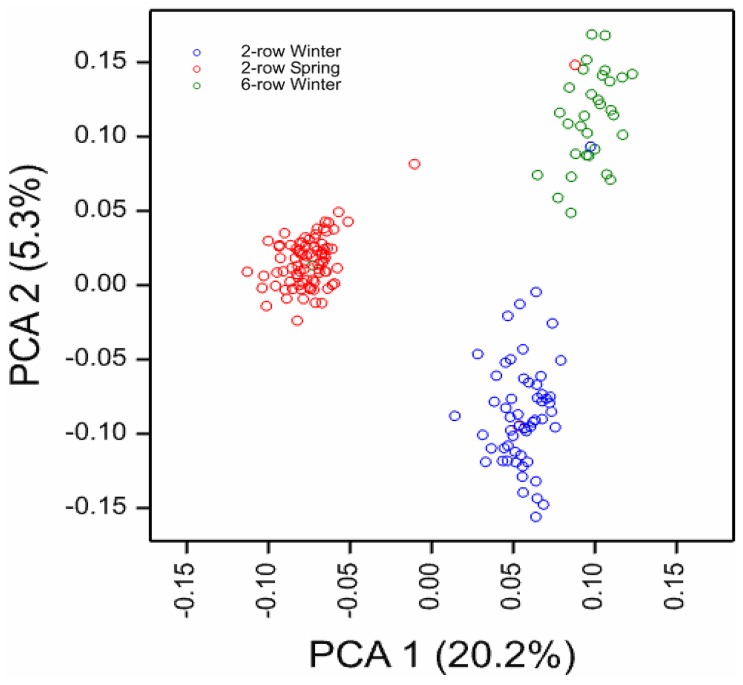
Scatter plot of the first two principal components show the distribution among the cultivars. Cultivars are classified by type: 2-row spring (blue), 2-row winter (red), and 6-row winter (green). The variance explained by each principal component is given in the axis heading.

The first three principal components were found to be significant factors by the eigen analysis, and they cumulatively explained 28% of the total variation (20.2% and 5.3% with the first and second axes, respectively). The plot of the first two principal components showed a clear division of the germplasm into three subpools, which largely coincided with the row number and seasonal habit (2-rowed spring, 2-rowed winter, and 6-rowed winter; Figure 2). The only exceptions were five cultivars, which clustered differently, according to what their *a priori* classifications would suggest. These included the 2-row spring varieties “Fergie” and “Phantom”, which were located in the 2- and 6-row winter pools, respectively; the 2-row winter variety “Cordoba”, which was grouped with the 6-row winter types; the 6-row winter variety “Tilia”, which was located in the 2-row spring pool; and the 2-row spring variety “Stella”, which appeared isolated between the 2-row spring and the 6-row winter pools. These results were consistent with results observed in other barley studies [45]. Based on this principal component analysis (PCA), we concluded that the first three principal components represented the major structure in the population. Therefore, we decided to use principal component scores as covariates in other models as an effective strategy to correct for population stratification (i.e., when assessing LD between markers, and when testing for associations between markers and traits).

**Figure 3:**
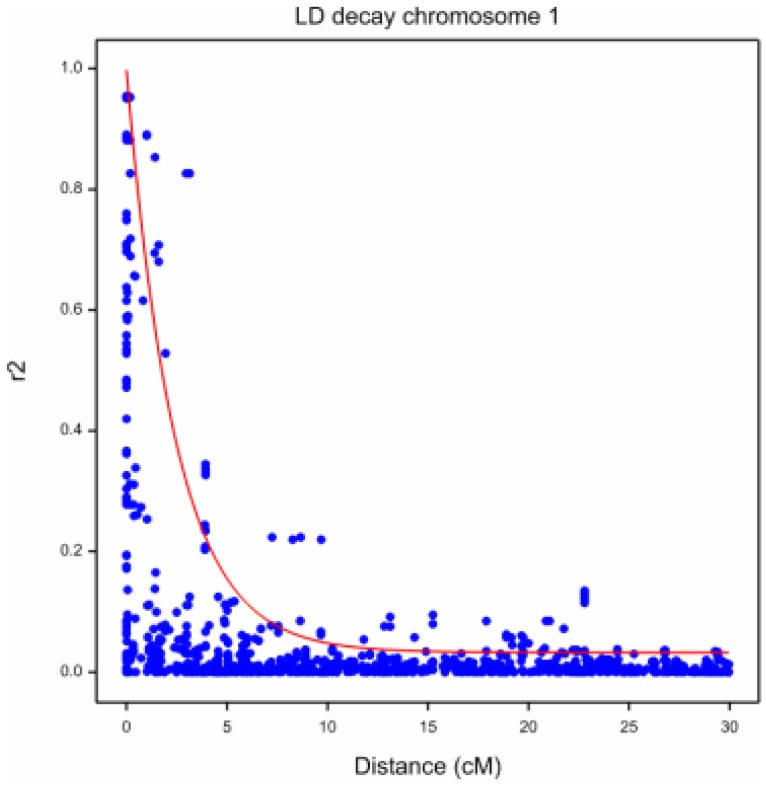
Intrachromosomal Linkage disequilibrium (LD)-decay between all pairs of DArT markers for chromosome 1H. LD between markers (*r* ^2^) is a function of marker distances (cM).

After correcting for population structure, the intrachromosomal LD was studied in all seven barley chromosomes by inspecting the plot of the associations between linked markers (r^2^ values) and their map distances, in cM (Figure 3; Figure S3). Taking a value of r^2^ = 0.20 as a strict threshold, based on the upper 0.95 quantile of the observed r^2^ values between unlinked markers, we found that the markers were, on average, in LD up to a distance of 5 cM. When we imposed the more liberal threshold of r^2^ = 0.10 (upper 0.80 quantile), the markers were, on average, in LD up to a distance of 10 cM.

In addition to assessing the marker density, the LD-decay information was used to define a Bonferroni-like multiple testing correction factor that we applied in the GWAS. The correction factor was defined as the total number of genome-wide independent tests, which was calculated as the number of chromosome blocks which were in LD, summed over all chromosomes. The corresponding correction factors were used for evaluating the significance (expressed as -log_10_ *P*) of MTAs. We used significance thresholds of -log_10_(*P*) >3.65 and –log_10_(*P*) >3.35, based on the strict (r^2^ = 0.20) and more liberal (r^2^ = 0.10) thresholds for LD, respectively. Cumulative p-values obtained by the naïve model and the MLM with correction for population structure and kinship by PCA were compared (Figure S4).

### Genome-wide association study (GWAS)

The inflation factors for all traits and models are shown in Table 3. As expected, a large inflation factor was observed for nearly all traits when the model did not account for genetic relatedness (naïve model). However, we observed that, even with the naïve model, the inflation factor was low for final attenuation (FiAt), TGW, raw protein in malt (M_RP), and beer color, which indicated that few strong associations were expected between the markers and those traits (as confirmed with the other models). The inflation factor fell substantially in all five models that accounted for genetic relatedness. The kinship model showed the steepest fall in inflation factor, with values very close to 1 (values below 1 indicated that the correction was too conservative). In models that used groups (based on population structure) and principal component scores to correct for genetic relatedness, the inflation factors decreased substantially, but not as much as the drop observed with the kinship model. Little difference was observed when the correction was considered a fixed or random term in a given model. However, on average, across all traits, the model that used principal component scores as fixed covariables performed slightly better (lower inflation factor) than the other models. Therefore, the following discussion is focused on the MTAs found with the model that used PCA scores as fixed terms.

**Table 3:** Genome-wide inflation factors of the naïve (uncorrected) model and five other models that account for genetic relatedness.

Trait	Naïve	Groups random	Groups fixed	PCA scores random	PCA scores fixed	Kinship
GY	8.27	1.17	1.21	1.33	1.38	1.12
TGW	2.29	1.46	1.46	1.54	1.55	1.10
HLW	3.01	1.55	1.53	1.46	1.50	1.02
KF	4.03	1.44	1.43	1.38	1.51	1.13
GF	6.91	1.85	1.82	1.57	1.73	1.20
SF <2.2 mm	7.05	1.45	1.46	1.42	1.44	1.02
SF_2.2–2.5 mm	5.09	1.31	1.28	1.34	1.33	0.10
SF_>2.8 mm	4.29	1.49	1.45	1.47	1.46	1.00
K_RP	11.98	1.94	1.87	1.93	1.80	1.31
M_RP	2.06	1.72	1.69	1.57	1.65	1.13
SolN	3.25	1.47	1.59	1.46	1.55	1.06
SolP	4.84	1.73	1.79	1.61	1.68	1.03
Visc	6.50	1.31	1.32	1.19	1.23	1.15
Col	2.09	1.44	1.42	1.46	1.42	1.06
Fria	8.89	1.84	1.86	1.69	1.75	1.07
VZ45	5.06	1.59	1.59	1.46	1.46	1.06
Extr	8.03	1.66	1.64	1.50	1.51	1.10
FiAt	1.38	1.38	1.73	1.38	1.36	1.01
MQI	6.29	1.85	1.87	1.65	1.71	1.11

**Figure 4:**
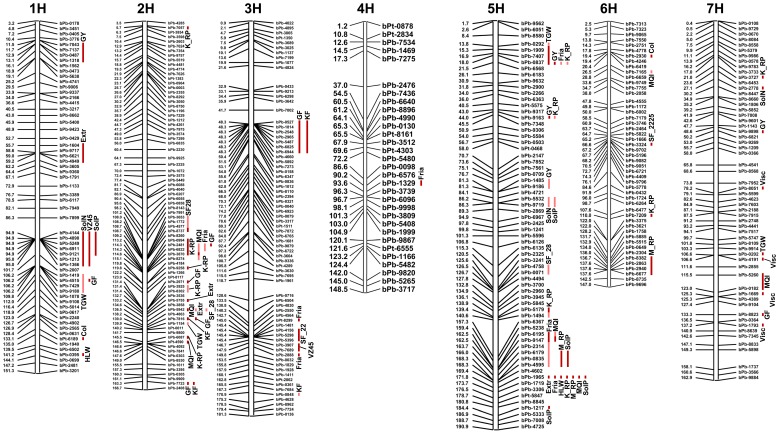
Barley consensus map with DArT markers significantly associated with kernel and malting quality traits. Only markers with the highest effect on a given chromosomal position are depicted. All MTAs that reflect kernel and malting quality parameters are defined either with a strict significance threshold = -log_10_(P)>3.65 (dark red) or a liberal significance threshold = -log_10_(P) <3.35 (light red). DArT = Diversity Array Technology, MTA = marker-trait association, GY = grain yield, TGW = thousand grain weight, HLW = hectoliter weight, KF = kernel formation, GF = glume fineness, SF = sieve fraction, K_RP = raw kernel protein content, M_RP = raw malt protein content, solN = soluble nitrogen, solP = soluble protein, Visc = viscosity, Col = color, Fria = friability, VZ45 = saccharification number VZ45°C, Extr = malt extract, FiAt = final attenuation, MQI = malting quality index.

With a threshold of –log_10_(P) >3.35, we identified 140 MTAs. With a more strict threshold of −log_10_(P) >3.65, we found 101 significant MTAs. These numbers are remarkably large, considering the relatively low sample size of this study (Table 4, Figure 4). We also observed an association between the heritability (h^2^) of the traits and the number of detected MTAs. More MTAs were found for high-heritability traits than for low-heritability traits. For example, kernel formation (KF) and glume fineness (GF) had nearly the highest h^2^ values (both 0.78; Table 1) and were associated with high numbers of markers (10 and 13, respectively; Table 4). In contrast, final attenuation had one of the lowest h^2^ values (Table 1), and it was not associated with any markers (no MTAs; Table 4).

**Table 4:** Number of significant (# sign) MTAs, based on high and low thresholds of significance, identified by applying the GWAS model with eigenvalues as fixed effects (fixed PCA score model).

Trait	# sign. MTAs	# sign. MTAs	# sign. MTAs	Chromosomes
	–log10(P) >3.65	3.35 < −log10(P) <3.65	−log10(P) >3.35	with MTAs
GY	6	2	8	1H, 5H^(2)^, 7H
TGW	3	5	8	1H, 5H^(2)^
HLW	2	0	2	1H, 5H
KF	9	1	10	2H^(2)^ , 3H
GF	13	0	13	1H, 2H^(2)^, 3H, 7H
SF_<2.2 mm	3	1	4	3H
SF_2.2–2.5 mm	1	0	1	6H
SF_>2.8 mm	1	5	6	2H
K_RP	11	4	15	2H^(3)^, 5H^(3)^, 6H, 7H
M_RP	8	0	8	5H, 6H
SolN	8	2	10	1H,7H
SolP	7	3	10	1H, 5H^(2)^
Visc	4	0	4	7H^(4)^
Col	2	0	2	1H, 6H
Fria	6	7	13	3H, 4H, 5H^(2)^
VZ45	5	4	9	1H, 3H
Extr	5	1	6	1H, 2H, 5H
FiAt	0	0	0	–
MQI	7	4	11	2H^(3)^, 5H^(2)^, 7H^(2)^
Total	101	39	140	

Markers located within 5 cM of each other were considered to be the same MTA. The superscript number shown in parentheses next to a chromosome number indicates the number of MTAs on that chromosome.

**Figure 5:**
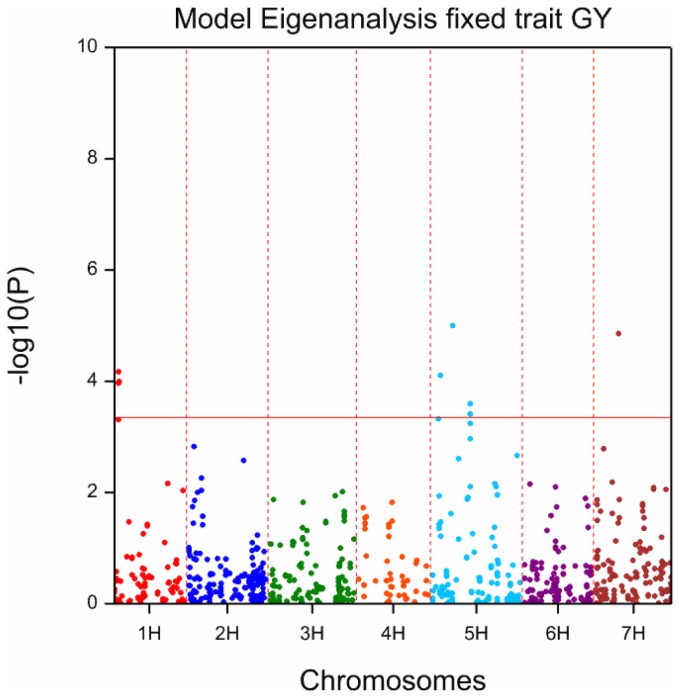
Manhattan plot for GWAS of grain yield (GY), considering eigenvalues (PCA scores) as fixed effects. The significance threshold is -log_10_(P) >3.65.

The complete list of MTAs is shown as supporting information in Table S2. Most of the 140 MTAs were observed on barley chromosomes 1H and 5H (Table 4 and Figure 4). Chromosome 5H clearly stood out, with about one third of the detected MTAs (41 out of 140). This was followed by chromosomes 1H and 2H with 30 and 28 MTAs, respectively. Only one MTA was detected on chromosome 4H (Table 4). The locations of markers and their associated traits are displayed in Figure 4 and summarized in Table 4 and Table 5. Some markers were associated with multiple traits. Most of the MTAs that were associated with multiple traits were located on chromosomes 1H, 2H, and 5H, and to some extent on chromosomes 3H and 7H. Some MTAs for different traits were co-localized within a small region of a chromosome (cluster). Most MTA clusters were located on 1H, 2H, 3H, and 5H (Figure 4). The region around 94.5 cM on chromosome 1H was tagged with many MTAs that were associated with multiple traits (SolN, SolP, and VZ45) consistent with the high correlations observed among these traits (Figure 1). The region between 110 and 165 cM on 2H was tagged with MTAs for several different traits, which indicated another hot spot relevant to malting and brewing quality. Furthermore, MTAs for GY, TGW, friability, and K_RP were located on chromosome 5H in the region between 13.8 and 18.0 cM. On the same chromosome, another dense concentration of MTAs was found in the region between 159 and 180 cM (Figure 4, Figure 5, and Figure S5). A summary of the common MTAs is provided as supporting information (Table S2). In addition, Table S2 shows the marker-allele substitution effects for all 140 MTAs.

**Table 5:** Genome-wide marker-trait associations (MTAs) detected at a significance threshold of –log_10_(P) >3.35 with the PCA scores fixed model are compared with known reference quantitative trait loci (QTLs) in the same chromosomal regions.

Chr	Chr-Pos [cM]	DArT marker	Traits with MTAs	Traits with Ref QTL	Position of Ref QTL [cM]	Designation of Ref QTL	Literature References
1H	11.5–13.1	bPb-7137	GY	GY	21.2	QYld.pil-1H	[12], [60], [61]
	58.7–59.2	bPb-6621, bPb-4949, bPb-9717	Extr	Extr, GY	53.5, 60.7, 63.9	QMe.StMo-1H.3, QMe.SlAl-1H	[60]–[65]
	–	–	–	Fria, MQI	58.7	QFRI1 1H gP68M59_200-Bmac90	[65]
		bPb-4144, bPb-4898,				QKp.HaMo-2H,	
	94.9	bPb-5249, bPb-6911,	SolN, SolP,	KF, Extr	92.6 – 100.8	QKp.nab-2H,	[62]
		bPb-9121, bPb-1213, bPb-1366	VZ45			QMe.StMo-1H.4	[66]
	106.2	bPb-1419, bPb-7429, bPb-9180	TGW	Extr	106.4	QMe.nab-1H.2	[63], [66]
				TGW,		QTw.HaMo-1H	
	106.2	bPb-4515	GF	HLW	106.4 – 126.7	QTw.HaMo-1H	[62], [67]
	133.1	bPt-6189, bPb-0395	Col, HLW	Yield	144.2	QYld.HaMo-1H.2	[30], [67]
2H	5.8	bPb-7057	K_RP	–	–	–	–
	108.1 – 108.7	bPb-3653, bPb-1772, bPb-8737	SF>2.8 mm	GY, TGW	92.2–100.8	QYld.StMo-2H.2, QTw.HaMo-2H, QTw.nab-2H, QTw.TyVo-2H.1	[62], [63], [66]–[68]
	113.2– 115.2	(bPb-0994), bPb-2481, bPb-6822, bPb-3870, (bPb-8274)	K_RP, GF, Fria, MQI	GPC (K_RP)	113.2, 114.4	bPb-0994, bPb-6822	[69]
	119.9	bPb-9258	K_RP	–	–	–	–
	131.5	bPb-2971, bPb-3925, bPb-8302	GF, K_RP	Yield	192.2	QYld.pil-2H.1	[12]
	133.3	bPb-5755	Extr	–	–	–	–
	136.6	bPb-5942	SF>2.8 mm, Extr, MQI	–	–	–	–
	139.0 – 139.9	bPb-7816, bPb-1154	GF, KF, TGW	Extr, TGW	138.2; 138.8	QMe.BlKy-2H.2, QTw.BlKy-2H.2	[62], [70]
	145.6 – 146.6	bPb-6087, bPt-4590	K_RP, MQI	solP	147.6	bPb-1986	[46]
	1633	bPb-7723	GF, KF	–	–	–	–
3H	48.3	bPb-0527, bPb-1814, bPb-2548, bPb-2965, bPb-5487, bPb-6825, bPb-6944	KF, GF	GY, MQ_RP, solN/solP	44.1–48.3	QYld.StMo-3H.1, QS/T.DiMo-3H	[62], [63], [71]–[74]
	145.1 – 145.5	bPt-8299, bPb-4156, bPb-5298, bPb-5396,	Fria, SF<2.2 mm	GPC (K_RP)	145.5	bPb-5298	[69]
	146.8, 147.9	bPb-3907, bPb-7689	VZ45	–	–	–	–
	149.8	bPb-2888, bPb-9599	KF, Fria	GPC (K_RP)	149.8	bPb-9599	[69]
	171.0	bPb-0848	KF	Yield	163.8–181.3	QYld.HaTR-3H	[75]
4H	93.6	bPb-1329	Fria	Yield, GPC (K_RP)	97.7	QYld.HaTR-4H; QGpc.HaTR-4H.2	[62], [75]–[77]
5H	13.8	bPb-0292	TGW	–	–	–	–
	15.4	bPb-1909	TGW	–	–	–	–
	18.0	bPb-0837, bPb-2872	GY, TGW, K_RP, Fria	–	–	–	–
	44.0	bPb-9163	GY, K_RP	FiAt, Fria, MQI	48.8–51.0	QEV3 5H gP69M61_211-gE32M58_387, QFRI2 5H cP70M48_294-cP68M59_571, QMQI4 5H cP70M48_294-cP68M59_571	[65]
	81.3	bPb-1485, bPb-9186	GY	GY	79.3	QPgw.BlKy-4H	[70]
	86.3	bPb-5532, bPb-9179	SolP, SolN	Extr	59.4–93.8	gE33M55_533	[65]
		bPb-0071, bPb-5179	SF>2.8 mm	Visc	132.1	QEv.HaTR-5H	[562]
	140.7	bPb-1494	K_RP	K_RP, GY, MQ_RP*,	139.4–140.7, 144.3*	QGpc.DiMo-5H.2	[12], [62]
	159.4	bPb-5238	SF>2.8 mm	GY	150.7	QYld.pil-5H.3, QYdp.S42IL-5H.	[12], [78]
	162.6	bPb-6195, bPb-9147	Fria, MQI	–	–	–	–
	166.1 & 168.3	bPb-6179 & bPb-0835, bPb-4595	M_RP, solP, Fria	Extr	169.4	QMe.DiMo-5H.3	[62], [71]
	171.9	bPb-1965	HLW, K_RP, M_RP, solP, Extr, Fria, MQI	HLW	173.2	QTw.HaTR-5H.2	[62], [75]
	184.4	bPb-1217	solP	SolP, solN/SolP	182.8	QS/T.HaMo-5H	[66], [67]
6H	19.4	bPb-2930	Col	Extr	28.4	QFcd.HaTR-6H	[62], [75], [76]
	26.5	bPb-7165	MQI	TGW, HLW, fine coarse difference (SF)	28.4	QTw.HaMo-6H.1, QFcd.HaTR-6H	[62] [62], [67], [75], [76], [79]
	68.2	bPb-9082	SF2.2–2.5 mm	Yield (GY)	68.5	QYld.StMo-6H, QTgw.S42IL-6H.	[63], [71], [72], [78]
	110.1	bPb-7209	K_RP	GY, TGW	97.9, 105.1	QYld.BlKy-6H, QTw.HaTR-6H	[62], [70], [75]
	136.7–137.7	bPb-8382, bPb-2863, bPb-2940¸ bPb-6677,	M_RP	Sol_Prot	140.8	QELG8 6H GBM1008–GBM1022	[65]
7H	21.1	bPb-3727	K_RP	TGW	17.0	QTw.BlKy-7H.1	[70]
	27.1	bPb-2778	solN	GF	28.4	QFcd.HaTR-6H	[62], [76]
	48.6	bPb-9898	GY	GY SF	35.2–48.6, 45.2	GYw1.2, QFcd.DiMo-7H	[62], [71], [76], [80]
	78.2	bPb-8051	Visc	K_RP	73.9	bPb-7952	[46]
	106.6	bPb-0202, bPb-4191	TGW, Visc	GY	106.6	QYld.pil-7H.2, QYdp.S42IL-7H.	[12], [78]
	115.6	bPb-5260	MQI	–	–	–	–
	123.1	bPb-0182	MQI	–	–	–	–
	125.4	bPb-1669	Visc	–	–	–	–
	133.4	bPb-8823	GF	TGW, SF		QTw.HaTR-7H.2, QFcd.HaTR-7H	[62], [75], [76]
	137.2	bPb-1793	Visc	GY	138.8	QYld.pil-7H.3	[12]

Abbreviations: Chr = chromosome; Chr-Pos = position of MTA on the chromosome; DArT: diversity array technology; Ref QTL = reference QTL; GY = grain yield, TGW = thousand grain weight, HLW = hectoliter weight, KF = kernel formation, GF = glume fineness, SF = sieve fraction, K_RP = raw kernel protein content, M_RP = raw malt protein content, solN = soluble nitrogen, solP = soluble protein, Visc = viscosity, Col = color, Fria = friability, VZ45 = saccharification number VZ45°C, Extr = malt extract, FiAt = final attenuation, MQI = malting quality index.

## Discussion

This study employed an association mapping approach to reveal the genetic basis of several kernel and malting quality parameters in barley. The MTAs identified in this study suggested that some genetic regions are highly important for breeding barleys with enhanced kernel and malting qualities. Some of the identified MTAs confirmed previously known QTLs [[12], [46], [60]–[80], but others were identified for the first time in this study (Table 5). Here, we discovered MTAs on all seven barley chromosomes. We found high MTA concentrations on chromosomes 1H, 2H, and 5H (Figure 4), which mostly represented former identified QTL-hotspots [62].

We discovered some novel genomic regions associated with malting quality on chromosomes 2H, 5H, 6H, and 7H. For example, we found associations for M_RP, SF (2.2–2.5 mm), and color on chromosomal regions of 6H that had not been identified before as QTLs. Furthermore, new MTAs were detected for K_RP, extract, KF, and GF, on chromosome 2H; for TGW, friability, and MQI on chromosome 5H; and for viscosity and MQI on chromosome 7H. Overall, the DArT markers located in previously described QTLs and the MTAs that we found were generally associated with the same or similar characteristics (Table 5). For example, the MTA found at 58.7–59.4 cM on chromosome 1H was associated with malt extract, viscosity, and friability, consistent with QTLs reported elsewhere [60]–[65]. Moreover, two strong QTLs for grain yield on chromosomes 1H and 7H coincided with those described previously [12], [60], [61] and [62], [71], [80], respectively. MTAs for GY and TGW on 5H and 6H were comparable to those detected in other studies [12], [78], [79]. Furthermore, the MTA found on 5H at 184.4 cM for SolP matched a QTL in this genomic region [66] and two other QTLs for yield on 7H [12]. Some of the MTAs that were associated with phenotypic characteristics also mapped to genomic regions close to QTLs associated with related traits. For example, the QTL QYld.StMo-3H.1 for GY [3], [63], [72]–[74] was located at 48.3 cM on chromosome 3H, where we found seven DArT markers that were strongly associated with kernel formation and glume fineness. On chromosome 2H, we detected some markers that were associated with these two traits in genomic regions that were previously reported to have an impact on yield parameters [62], [66]–[68]. We also discovered two DArT markers, bPb-0994 and bPb-6822 (located on chromosome 2H at 113.2 and 114.4 cM, respectively) associated with grain protein content, which were also found in another study [69]. We also found that the marker bPb-0994, located on chromosome 2H, was highly significant for kernel raw protein content. This marker was previously shown to be significant for grain protein content [69], in addition to three markers on 2H and 3H. Two other DArT markers on chromosome 3H, which were associated with grain protein content [69], were related to other traits in our study, including sieve fraction >2.2 mm (bPb-5298 at 145.5 cM) and friability (bPb-9599 at 149.8 cM), because we used a different germplasm.

It was not always straightforward to make comparisons with other known QTLs reported in the literature, because different studies used different reference maps, marker types, germplasms, experimental sites, and trait measuring protocols [46], [62], [81]–[86]. For example, we did not find MTAs identical to those found by Beattie et al. 2010 [46], because different germplasms were studied (North American *vs*. European material). A similar explanation can account for differences in GWAS that mapped GY in landraces cultivated in the high- and low-yield environments of Spain and Syria, respectively [40]. Only one of their associated DArT markers was also detected in our European elite germplasm (bPb-9163 on 5H). Again, this discrepancy was probably caused by the lack of correspondence between the different genetic backgrounds used by Pswarayi et al. (2008) [40] and our study. An association study with kernel quality parameters in a restricted subset of 101 almost identical winter barley varieties (48 2-row and 61 6-row types) was performed based on Illumina-SNP-markers [83]. Only the MTAs that we primarily associated with grain yield and hectoliter weight on chromosomes 1H and 5H matched the findings in that study [83]. Other barley association panel results that were based on different marker systems and germplasm pools (e.g., the Barley CAP germplasm) [87]–[88] also showed little congruence with our results.

Genetic correlations among traits typically result from either pleiotropic or tightly-linked QTLs. In the present study, we found many MTAs for different traits that co-localized to a single chromosomal region. This co-localization may drive genetic correlations among barley quality traits. In particular, we found clusters of MTAs for malting quality traits on chromosomes 1H, 2H, and 5H, which pointed to hot spots for barley quality. This was consistent with findings from Szücs et al. 2009 [62] and with another study that reported evidence of multilocus clusters that may regulate or control barley malting characteristics [71].

In general, our findings were consistent with the literature and corresponded well with the observed correlations among traits [75], [77], [87]–[94]. Most co-localized MTAs represented traits with high phenotypic correlations. For example, MTAs that correlated with the malting quality parameters, SolP, SolN, and VZ45, were detected in the same region on 1H (and 5H). This information may provide valuable guidance for understanding a multivariate response to a protocol designed to select for these traits.

Some traits, like MQI, friability, and extract are genetically correlated with each other. This correlation was reflected in our results by the finding that these traits were significantly associated with the same markers. Traits such as viscosity and friability or final attenuation, VZ45, and extract interact to define malting properties, which contribute to important phenotypic effects. It is crucial to be aware of these interactions to understand the trade-offs implied in the optimization of cultivars.

For example, breeders should be aware that high grain protein concentration is associated with low levels of malt extract. High grain protein increases the likelihood of a chill haze in beer, and barleys with low grain protein concentrations are more economically efficient in the malting process [90]. High protein reduces efficiency, because the grain takes up water slowly and unevenly during the germination process. In addition to producing low levels of malt extract, the resulting beer has a longer filtration time, develops cloudiness, and possesses a shorter shelf life. On the other hand, insufficient levels of grain protein may limit the growth of yeast during the fermentation process; it also reduces the stability of the beer head because the beer foam cannot cling effectively to the side of the glass. Consequently, maltsters prefer a GPC close to 10.5% [90]. It behooves the breeder to know which parameters are correlated, because these parameters require balancing to achieve the optimal outcome.

## Conclusions

The current work contributed to the understanding of the genetic basis of kernel and malting qualities in barley. The use of a broad phenotypic data collection that spanned a long time range and several locations provided a means to de-emphasize environmental effects on barley trait expression. We found that combining this historical phenotypic dataset with high-density, low-cost markers such as DArTs facilitated the discovery of new MTAs for barley. As shown previously, we found that association mapping was a powerful, promising approach for dissecting the complexities of malting and brewing qualities in barley. In addition to confirming various known QTLs, we identified some new MTAs; e.g., markers for MQI and viscosity. The MTAs identified in this study will be useful for selecting favorable genotypes in this germplasm that can be used to develop improved barley varieties. The findings of this study should be validated in future field experiments. Our research demonstrated the advantage of combining more than 20 years of expensive phenotyping information with high-density, low-cost marker technology.

## Materials and Methods

### Germplasm and phenotypic data

A set of 174 European barley cultivars that included 85 two-rowed spring and 89 winter types (57 two-rowed and 32 six-rowed) were included in this study. Historical phenotypic data were available for all 174 cultivars in the annual statistical reports from the German Brewery Association (http://www.braugerstengemeinschaft.de). The historical data were collected from 1985 to 2007 and stored in a database called “MetaBrew” [95]. The following nine agronomic traits were considered: grain yield (GY), thousand grain weight (TGW), hectoliter weight (HLW), kernel formation (KF), glume fineness (GF), three sieve fractions (SF), and raw kernel protein content (K_RP). Ten malting quality traits were also considered: raw malt protein content (M_RP), soluble nitrogen (solN), soluble protein (solP), viscosity (Visc), color (Col), friability (Fria), saccharification number VZ45°C (VZ45), malt extract (Extr), final attenuation (FiAt), and malting quality index (MQI). Malting quality parameters were assessed with standard procedures recommended by the European Brewery Convention (EBC) and the ‘Mitteleuropäische Brautechnische Analysenkommission (MEBAK)’.

### Genotyping with DArT markers

Seeds for all cultivars were obtained from the breeders. Seeds were grown into young plantlets. Leaf material was harvested from five to six seedlings that were 10 days old. The material was bulked, and genomic DNA was extracted according to the requirements of Triticarte Pty. Ltd. (Canberra, Australia), as described previously [25], [26]. A dense, whole genome scan was performed with Diversity Array Technology (DArT), which generated 1,088 mapped and 774 unmapped biallelic markers for this population, according to the published DArT consensus map [37]. The locus designations used by Triticarte Pty. Ltd. were adopted in this study, and DArT markers were named with the prefix “bPb,” followed by a unique numerical identifier. We removed markers with minor allele frequencies less than 0.05. Then, a set of 839 mapped DArT markers was selected for the GWAS to provide coverage that was evenly distributed over the seven barley linkage groups (Figure S2).

### Analysis of phenotypic data

Our objective was to concentrate on major, stable MTAs. Therefore, we calculated cultivar-adjusted means over locations and years for each of the traits. Prior to the analysis, traits that were expressed in percentages were log-transformed, such as sieve fraction (SF) and raw protein in kernel or malt (K_RP, M_RP). The following mixed model was used to estimate adjusted cultivar means (random terms underlined):
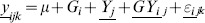


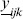
 is the observation of the *i^th^* cultivar, in the *j^th^* year, and the *k^th^* replicate (location) nested within year *j*; *µ* is an intercept; *G_i_* is the fixed cultivar effect; 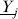 is the random year effect, where; 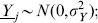 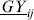 is the interaction between the cultivar and the year, where; 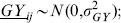 and 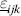 is a residual term, where; 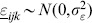. Note that, because locations are considered replications within a year, location effects (and corresponding interactions with cultivars) do not appear explicitly in the statistical model, but are pooled within the residual term, 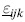. Evaluating locations as replicates was justified in this type of trial network, because all testing sites were mainly selected to represent the same target production environment. The best linear unbiased estimates (BLUEs) obtained from this model were used in the subsequent GWAS (Table S1).

### Analysis of linkage disequilibrium (LD) between markers

According to previous studies [96], [97], the LD between every pair of markers (*m, n*) in the same linkage group was assessed with the following statistical model:
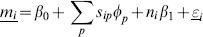
where; 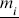 and *n_i_* are the scores of markers, *m* and *n*, of genotype *i* (with values –1 or 1 for either of the two homozygous genotypes); *S_ip_* denotes the scores of the first *p* principal components from an eigenanalysis (singular value decomposition of the molecular marker matrix), as described in [53]. This term represents the effect of population structure. The magnitude of the LD between the markers was assessed by the partial *r^2^* associated with the 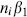 term. An empirical threshold for LD was determined by randomly sampling 1000 pairs of independent markers (i.e., markers known to map to different linkage groups). Two thresholds were used: one was strict, based on the upper 95% quantile of the distribution of *r^2^* values and the other was more liberal, based on the upper 80% quantile of the observed *r^2^* values. To assess how far the LD extended on a particular chromosome, we used the intersection of the threshold *r^2^* with a 95% quantile non-linear regression line fitted to the observed *r^2^* values on the particular chromosome. The non-linear quantile regression fitting was based on the method of Koenker & D’Orey [98], which has been implemented in GenStat 16 software [99]. The strict threshold was used to define a lower limit of the LD extension and the liberal threshold used to define an upper limit of the LD extension.

In turn, the LD-decay information for each chromosome was used to define a multiple testing correction threshold for the GWAS, as described previously [96]. This approach was based on a Bonferroni correction, but instead of using all markers as the denominator, it used the number of effective (independent) tests performed genome-wide. We defined the number of independent tests as 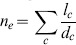, where; *l_c_* is the length in cM of chromosome *c*, and *dc* is the extension of LD for chromosome *c*, and calculated the P-value significance threshold, as follows (on a log scale) where; P^*^ is the genome-wide threshold level (set as 0.05 in our study):
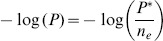



### Genome-wide marker-trait association analysis (GWAS)

GWAS was performed with models that accounted for the genetic relatedness between varieties. Genetic relatedness was expressed in several alternate ways, including the realized kinship (model K), a group factor, based on population structure (fixed or random group models), or a set of individual principal components scores that served as fixed or random covariables in the model (fixed or random PCA score models, respectively). We also used a model that did not include a correction for genetic relatedness (naïve model).
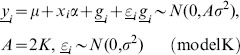





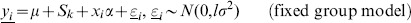





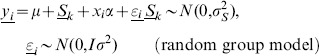





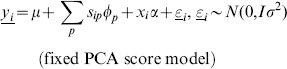





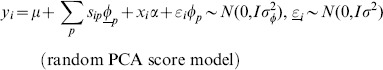





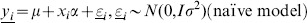



In the models above, *µ* is a constant (intercept); *x_i_* is a marker covariate with values −1 or 1 to denote one of the two homozygous marker genotypes; α is the marker effect; 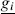 is a random polygenic effect; is the fixed group effect (random when underlined); *si_p_* denotes the scores of the first *p* principal components, and 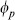 is the associated fixed effect (random when underlined).

The significance of each MTA was assessed with the Wald test, and results are expressed with the associated *P*-values on a –log_10_ scale. The performances of the different models were compared by their inflation factors. We focused the discussion of significant MTAs on results from the model that performed best (fixed PCA score model).

All models were fitted with GenStat version 16 [99] with the available features for LD mapping. The mixed linear model (MLM) was fitted with the residual maximum likelihood (REML) method. Graphical mapping of the most significant MTAs was performed with QGene version 4.3.7 [100].

### Comparison with known QTLs

For comparisons between significantly-associated DArT markers and known QTLs, the marker and chromosome position information from GrainGenes (http://www.graingenes.org) and from Barley World (http://www.barleyworld.org/) were compared to the reference DArT map created previously [37]. Some marker-associated traits assessed in this study were similar to those identified with known QTLs reported for barley in the GrainGenes database or literature. When the trait designation was missing, but similar, or limited information was available, results were compared between traits with similar features. For example, in some cases, HLW was compared to test weight; KF was compared to kernel length and plumpness; kernel weight was compared to TGW; plan test weight was compared to yield; friability was compared to milling energy and malt tenderness; and SolP was compared to the ratio of soluble/total protein ( = Kolbach index).

## Supporting Information

Figure S1 **Histograms of the phenotypic trait distribution among cultivars.** GY = grain yield, MY = marketable yield, TGW = thousand grain weight, HLW = hectoliter weight, KF = kernel formation, GF = glume fineness, SF = sieve fraction, K_RP = raw kernel protein content, M_RP = raw malt protein content, solN = soluble nitrogen, solP = soluble protein, Visc = viscosity, Col = color, Fria = friability, VZ45 = saccharification number VZ45°C, Extr = malt extract, FiAt = final attenuation, MQI = malting quality index.
(ZIP)

Figure S2 **Chromosomal distribution of the 839 DArT markerDArT markers used for the genome-wide association analysis.** Distances are given in cM.
(TIF)

Figure S3 **Intrachromosomal LD-decay between all pairs of DArT markers shown for each barley chromosome, 1H to 7H, after correcting for population structure.**
(TIF)

Figure S4 **Comparison of P-values obtained by applying the naïve model (blue line) and the mixed linear model (MLM).** The MLM incorporates corrections for population structure and kinship, based on PCA scores (red line). The comparison permits a check of the quality of the association results depicted for four traits (a) Grain yield (GY), (b) hectoliter weight (HLW), (c) kernel formation (KF), and (d) thousand grain weight (TGW).
(ZIP)

Figure S5 **Manhattan plots show GWAS results from the PCA-corrected model for all kernel and malting parameters.** GY = grain yield, MY = marketable yield, TGW = thousand grain weight, HLW = hectoliter weight, KF = kernel formation, GF = glume fineness, SF = sieve fraction, K_RP = raw kernel protein content, M_RP = raw malt protein content, solN = soluble nitrogen, solP = soluble protein, Visc = viscosity, Col = color, Fria = friability, VZ45 = saccharification number VZ45°C, Extr = malt extract, FiAt = final attenuation, MQI = malting quality index.
(ZIP)

Table S1 **Summary of phenotypic parameters for all 174 cultivars.** BLUES and means are shown for the breeder, origin, seasonal habit (SH), including spring (S) or winter (W), row number (RN), and phenotypic traits. Abbreviations are: BLUES = best linear unbiased estimators, GY = grain yield, MY = marketable yield, TGW = thousand grain weight, HLW = hectoliter weight, KF = kernel formation, GF = glume fineness, SF = sieve fraction, K_RP = raw kernel protein content, M_RP = raw malt protein content, solN = soluble nitrogen, solP = soluble protein, Visc = viscosity, Col = color, Fria = friability, VZ45 = saccharification number VZ45°C, Extr = malt extract, FiAt = final attenuation, MQI = malting quality index; Sheet 1: The average BLUES (best linear unbiased estimators) for the nine kernel traits and ten malting quality traits considered in this genome-wide association study. Each cultivar represents the average of several accessions of the same variety. Sheet 2: Individual accessions for each cultivar variety; the location of each accession is given with the BLUES (best linear unbiased estimators) for each trait. Sheet 3: Phenotypic means for each cultivar across all accessions. Group assignments indicate the population structures.
(XLSX)

Table S2 **Allele frequencies for DArT markers and marker effects on 19 phenotypic parameters based on MTAs identified in the GWAS in barley.** Results are shown for MTAs identified with strict (-log_10_(P) >3.65) or liberal (-log_10_(P) >3.35) significance thresholds.
(XLSX)
