# Supplementary figures and images for: Genome-Wide Association Mapping for Kernel and Malting Quality Traits Using Historical European Barley Records
Source: PLoS One. 2014 Nov 5;9(11):e110046. doi: 10.1371/journal.pone.0110046 (PMC4221631; doi:10.1371/journal.pone.0110046)

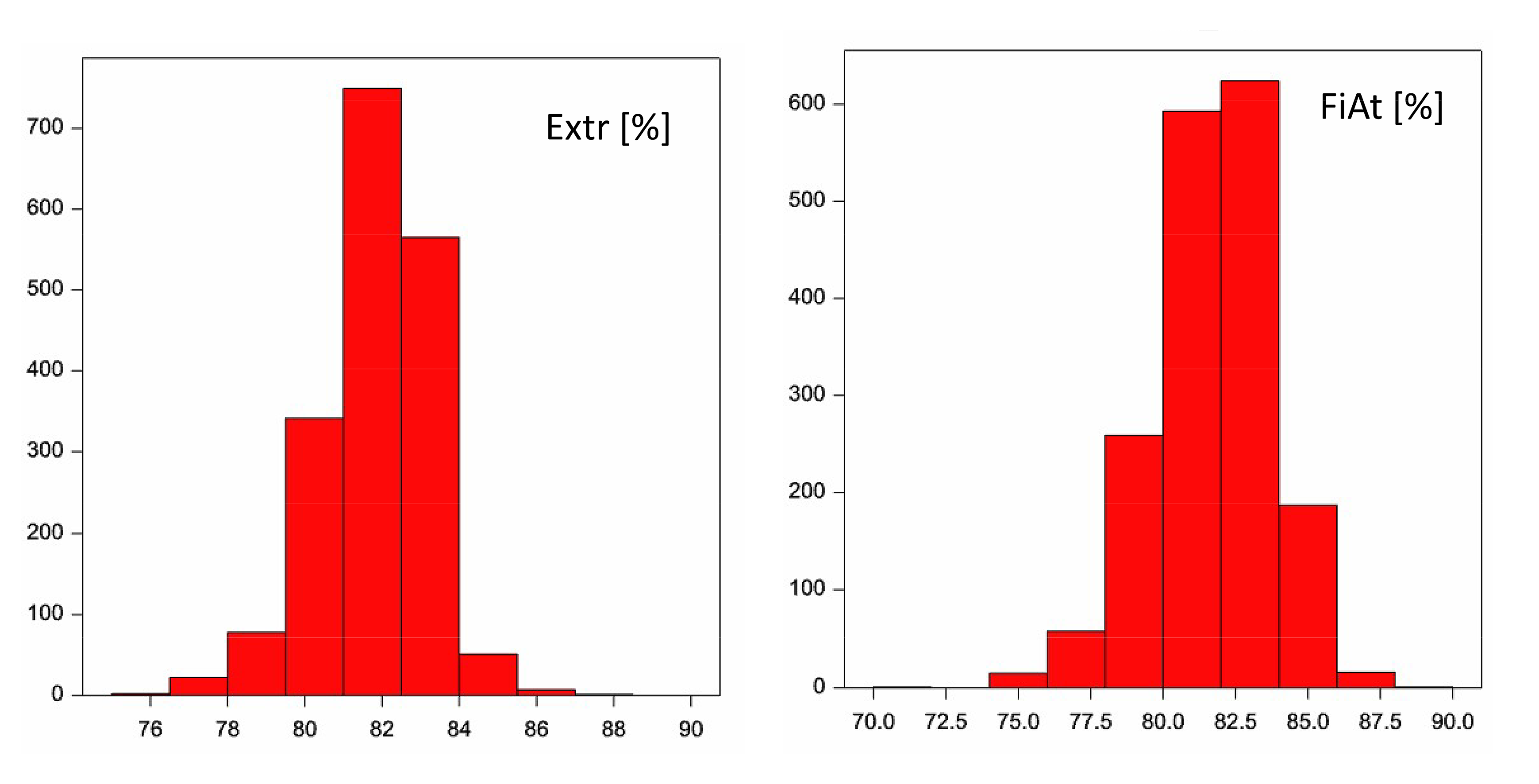

Supplement: Figure S1 — Histograms of the phenotypic trait distribution among cultivars. GY = grain yield, MY = marketable yield, TGW = thousand grain weight, HLW = hectoliter weight, KF = kernel formation, GF = glume fineness, SF = sieve fraction, K_RP = raw kernel protein content, M_RP = raw malt protein content, solN = soluble nitrogen, solP = soluble protein, Visc = viscosity, Col = color, Fria = friability, VZ45 = saccharification number VZ45°C, Extr = malt extract, FiAt = final attenuation, MQI = malting quality index. (ZIP) [file pone.0110046.s001.zip › Suppl_Fig_1-9_LZW_600dpi_.tif]

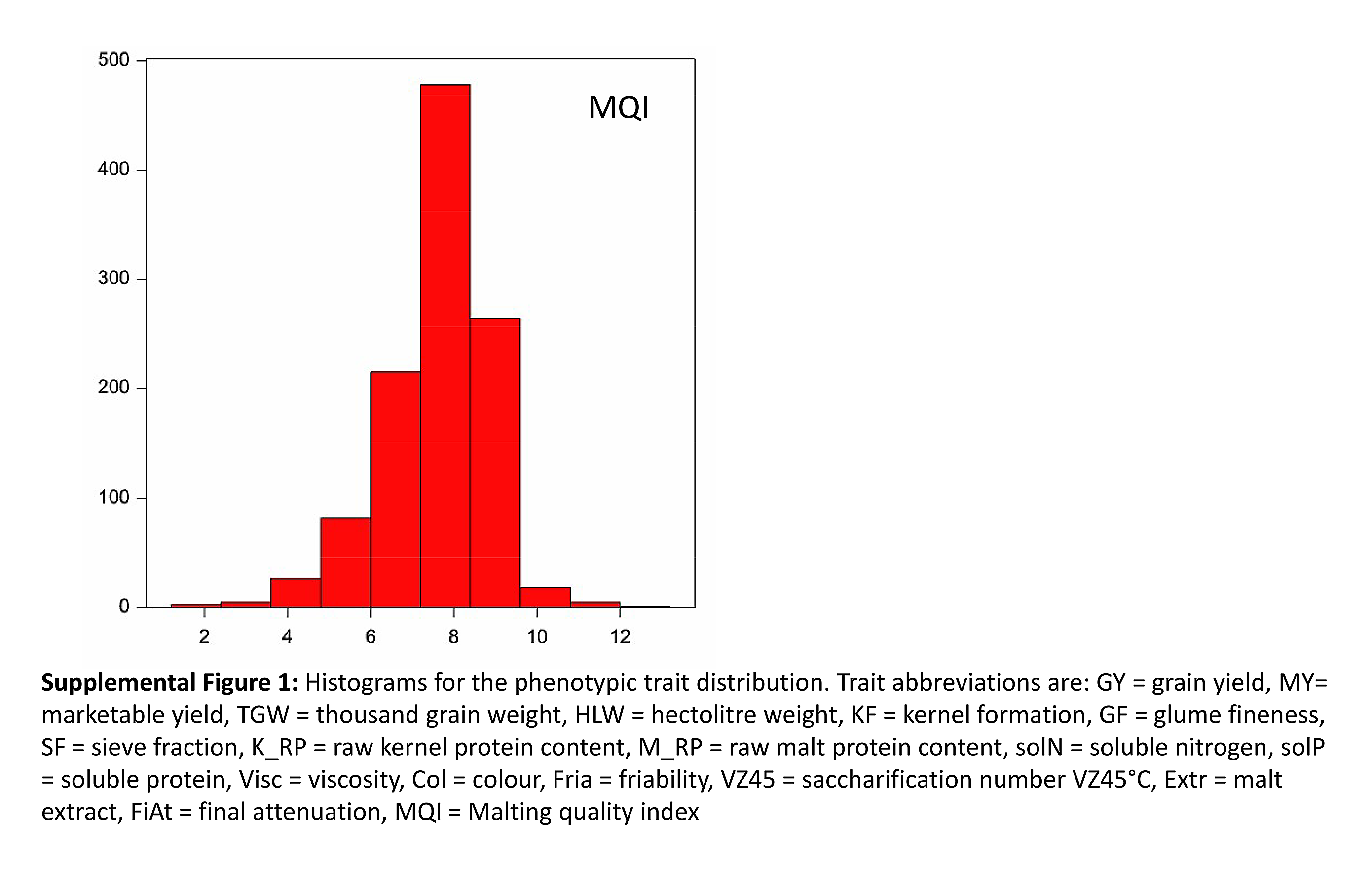

Supplement: Figure S1 — Histograms of the phenotypic trait distribution among cultivars. GY = grain yield, MY = marketable yield, TGW = thousand grain weight, HLW = hectoliter weight, KF = kernel formation, GF = glume fineness, SF = sieve fraction, K_RP = raw kernel protein content, M_RP = raw malt protein content, solN = soluble nitrogen, solP = soluble protein, Visc = viscosity, Col = color, Fria = friability, VZ45 = saccharification number VZ45°C, Extr = malt extract, FiAt = final attenuation, MQI = malting quality index. (ZIP) [file pone.0110046.s001.zip › Suppl_Fig_1-10_LZW_600dpi_.tif]

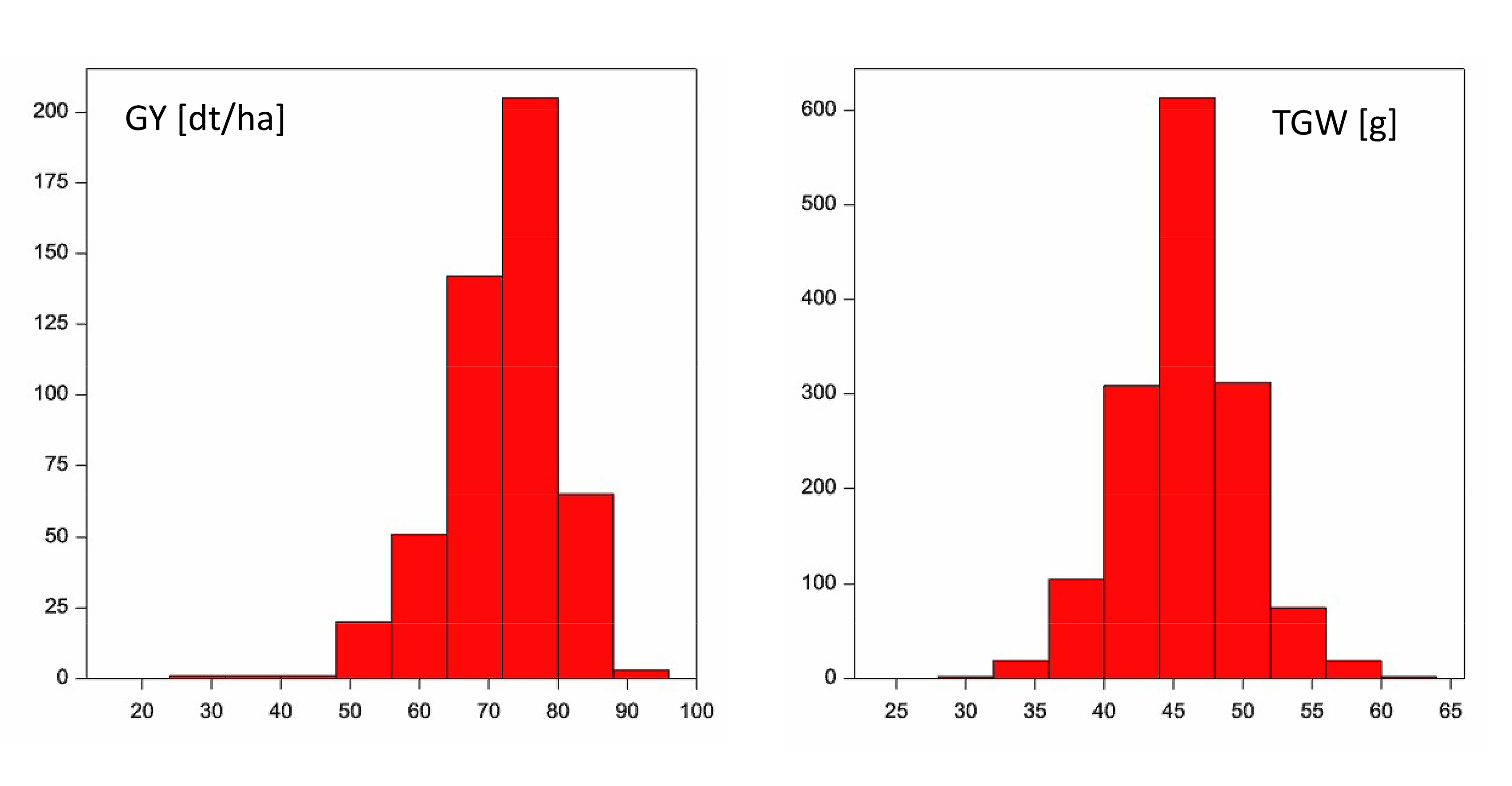

Supplement: Figure S1 — Histograms of the phenotypic trait distribution among cultivars. GY = grain yield, MY = marketable yield, TGW = thousand grain weight, HLW = hectoliter weight, KF = kernel formation, GF = glume fineness, SF = sieve fraction, K_RP = raw kernel protein content, M_RP = raw malt protein content, solN = soluble nitrogen, solP = soluble protein, Visc = viscosity, Col = color, Fria = friability, VZ45 = saccharification number VZ45°C, Extr = malt extract, FiAt = final attenuation, MQI = malting quality index. (ZIP) [file pone.0110046.s001.zip › Suppl_Fig_1-1_LZW_600dpi_.tif]

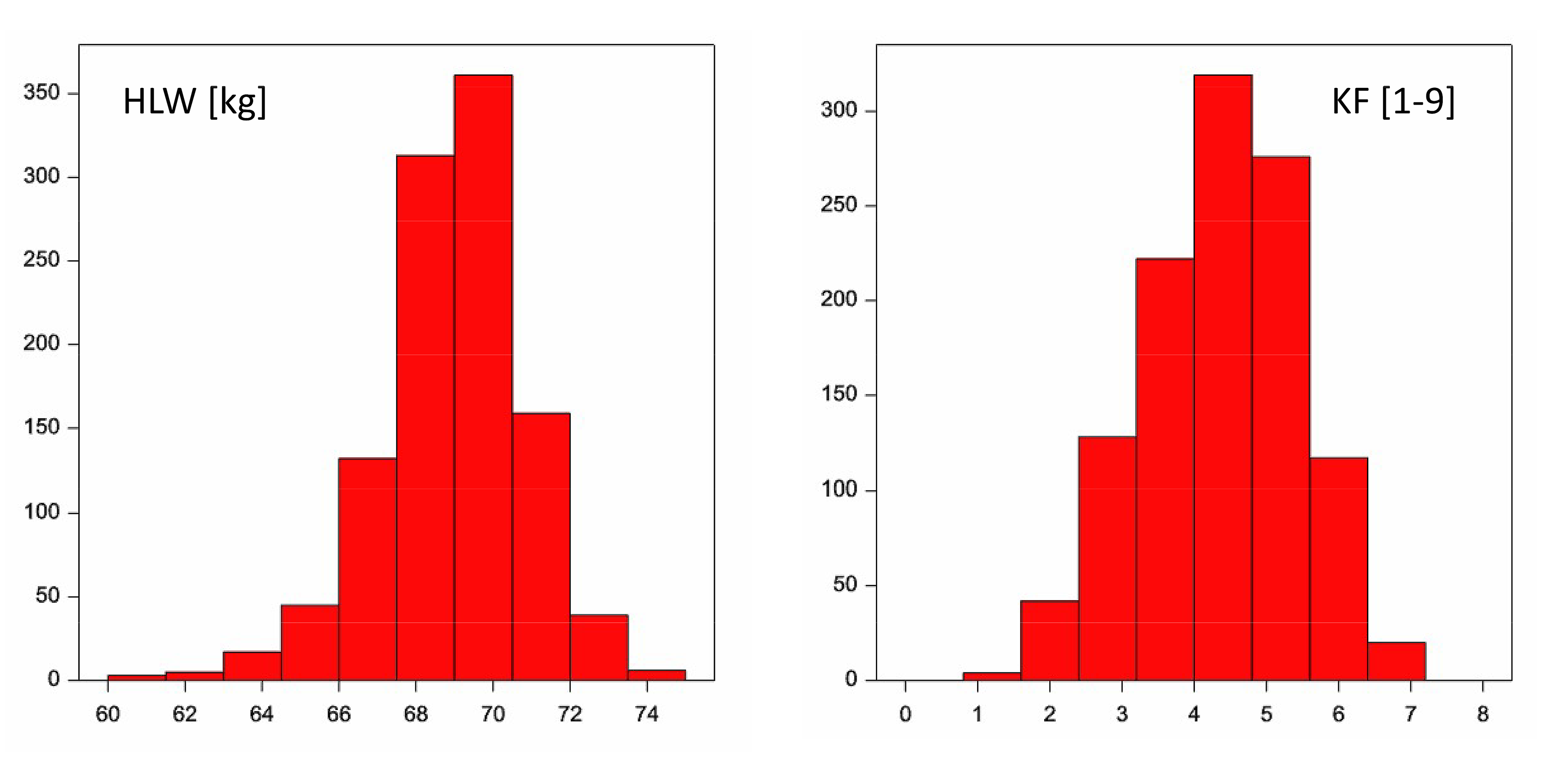

Supplement: Figure S1 — Histograms of the phenotypic trait distribution among cultivars. GY = grain yield, MY = marketable yield, TGW = thousand grain weight, HLW = hectoliter weight, KF = kernel formation, GF = glume fineness, SF = sieve fraction, K_RP = raw kernel protein content, M_RP = raw malt protein content, solN = soluble nitrogen, solP = soluble protein, Visc = viscosity, Col = color, Fria = friability, VZ45 = saccharification number VZ45°C, Extr = malt extract, FiAt = final attenuation, MQI = malting quality index. (ZIP) [file pone.0110046.s001.zip › Suppl_Fig_1-2_LZW_600dpi_.tif]

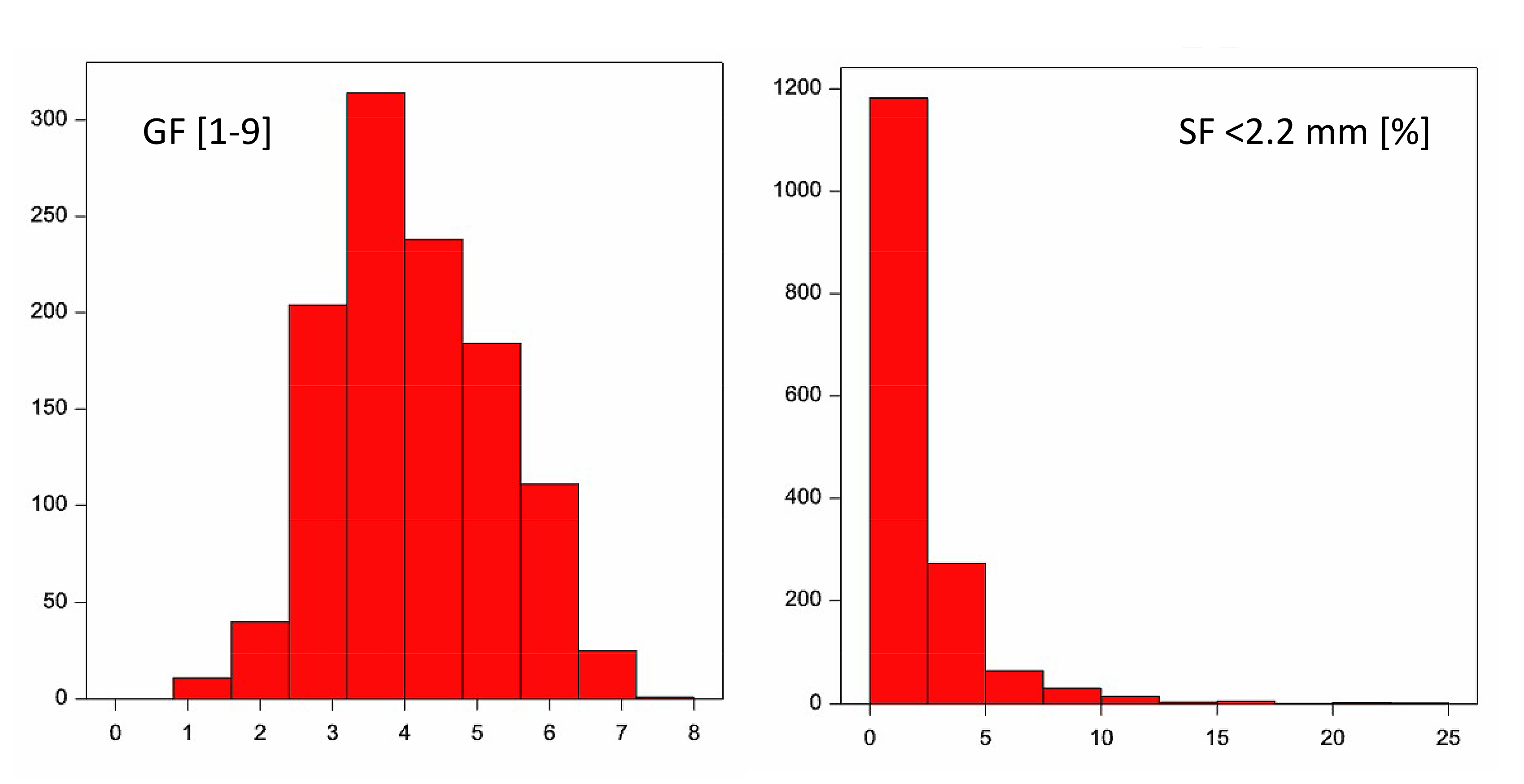

Supplement: Figure S1 — Histograms of the phenotypic trait distribution among cultivars. GY = grain yield, MY = marketable yield, TGW = thousand grain weight, HLW = hectoliter weight, KF = kernel formation, GF = glume fineness, SF = sieve fraction, K_RP = raw kernel protein content, M_RP = raw malt protein content, solN = soluble nitrogen, solP = soluble protein, Visc = viscosity, Col = color, Fria = friability, VZ45 = saccharification number VZ45°C, Extr = malt extract, FiAt = final attenuation, MQI = malting quality index. (ZIP) [file pone.0110046.s001.zip › Suppl_Fig_1-3_LZW_600dpi_.tif]

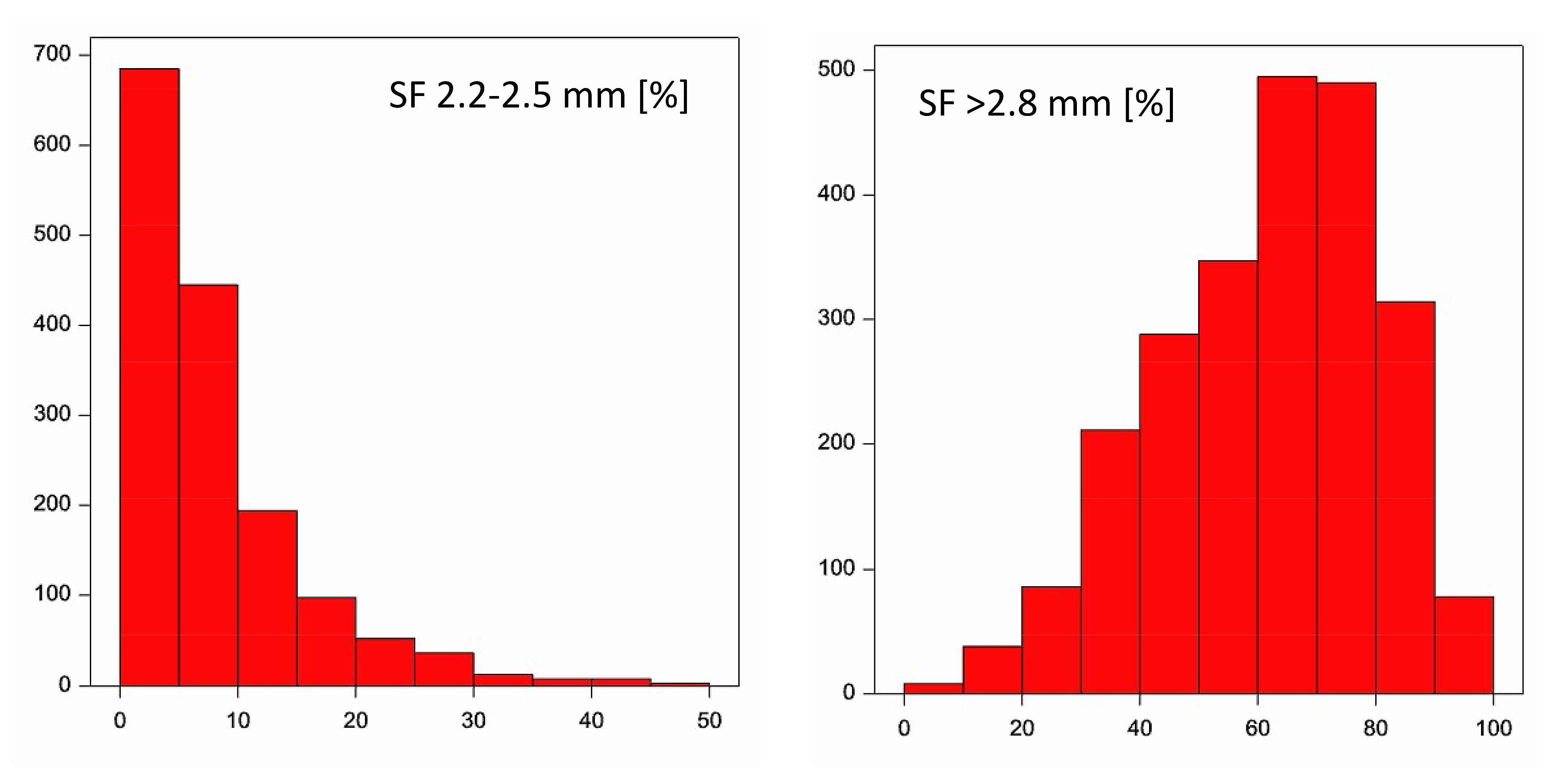

Supplement: Figure S1 — Histograms of the phenotypic trait distribution among cultivars. GY = grain yield, MY = marketable yield, TGW = thousand grain weight, HLW = hectoliter weight, KF = kernel formation, GF = glume fineness, SF = sieve fraction, K_RP = raw kernel protein content, M_RP = raw malt protein content, solN = soluble nitrogen, solP = soluble protein, Visc = viscosity, Col = color, Fria = friability, VZ45 = saccharification number VZ45°C, Extr = malt extract, FiAt = final attenuation, MQI = malting quality index. (ZIP) [file pone.0110046.s001.zip › Suppl_Fig_1-4_LZW_600dpi_.tif]

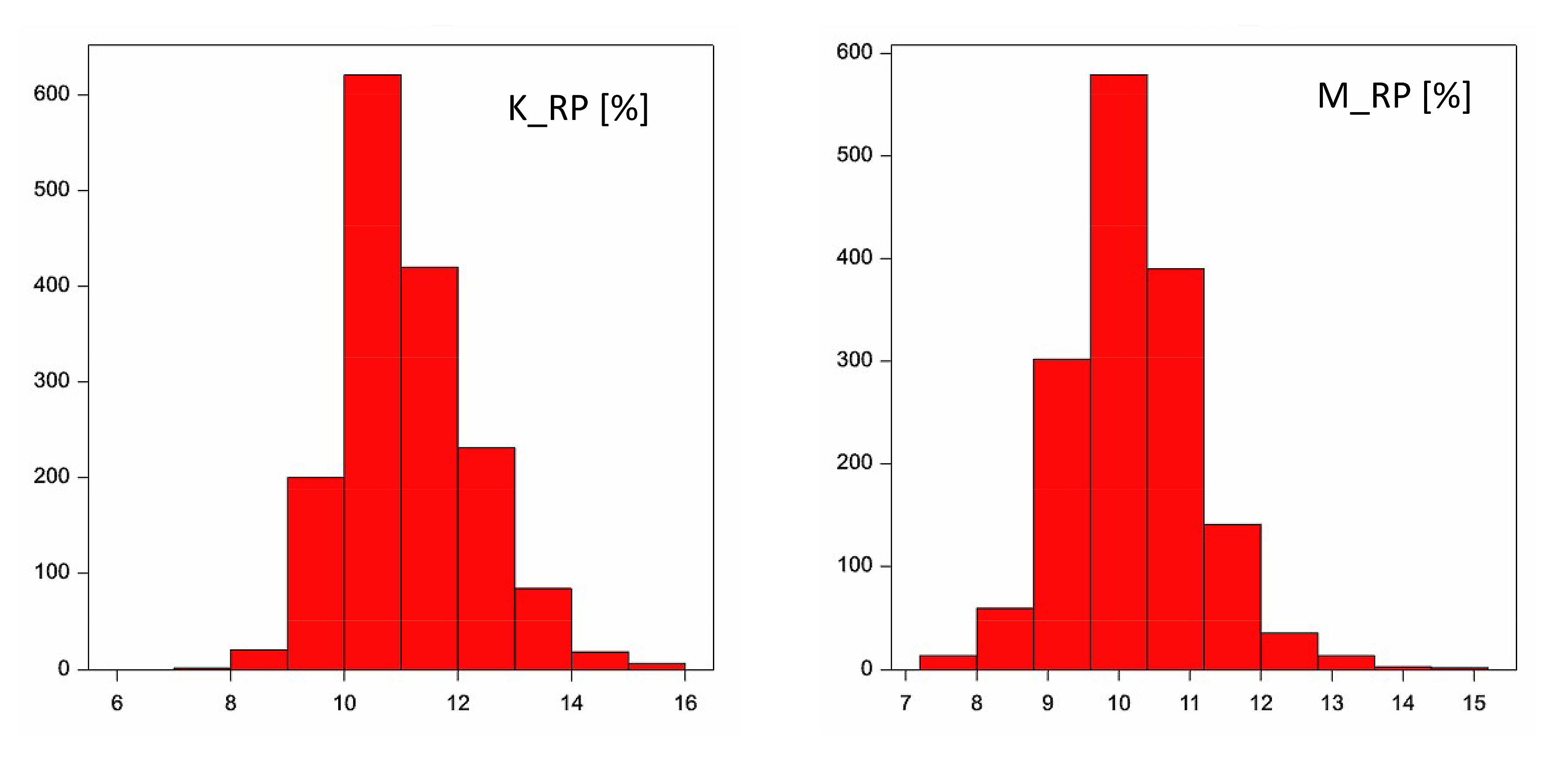

Supplement: Figure S1 — Histograms of the phenotypic trait distribution among cultivars. GY = grain yield, MY = marketable yield, TGW = thousand grain weight, HLW = hectoliter weight, KF = kernel formation, GF = glume fineness, SF = sieve fraction, K_RP = raw kernel protein content, M_RP = raw malt protein content, solN = soluble nitrogen, solP = soluble protein, Visc = viscosity, Col = color, Fria = friability, VZ45 = saccharification number VZ45°C, Extr = malt extract, FiAt = final attenuation, MQI = malting quality index. (ZIP) [file pone.0110046.s001.zip › Suppl_Fig_1-5_LZW_600dpi_.tif]

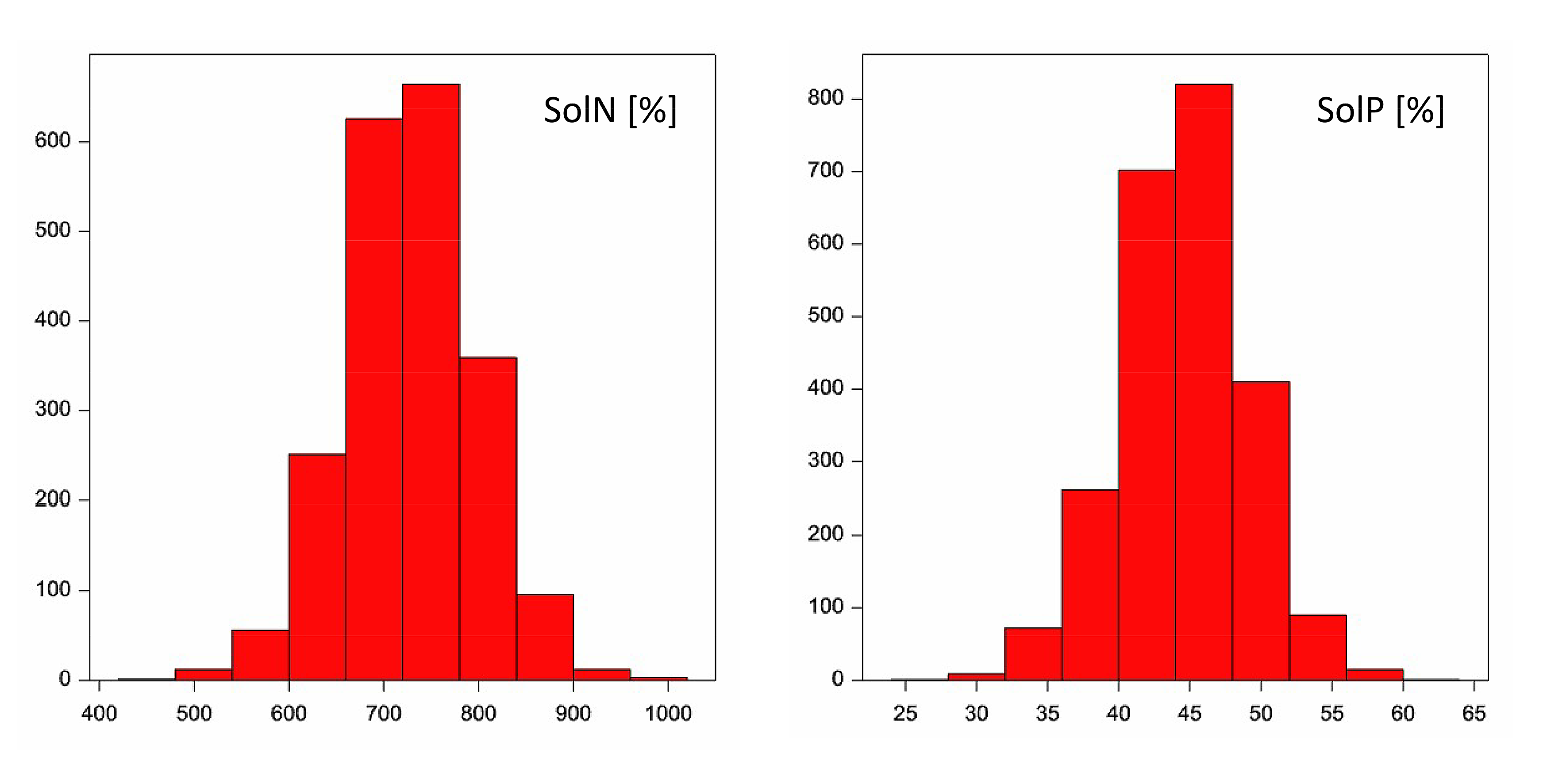

Supplement: Figure S1 — Histograms of the phenotypic trait distribution among cultivars. GY = grain yield, MY = marketable yield, TGW = thousand grain weight, HLW = hectoliter weight, KF = kernel formation, GF = glume fineness, SF = sieve fraction, K_RP = raw kernel protein content, M_RP = raw malt protein content, solN = soluble nitrogen, solP = soluble protein, Visc = viscosity, Col = color, Fria = friability, VZ45 = saccharification number VZ45°C, Extr = malt extract, FiAt = final attenuation, MQI = malting quality index. (ZIP) [file pone.0110046.s001.zip › Suppl_Fig_1-6_LZW_600dpi_.tif]

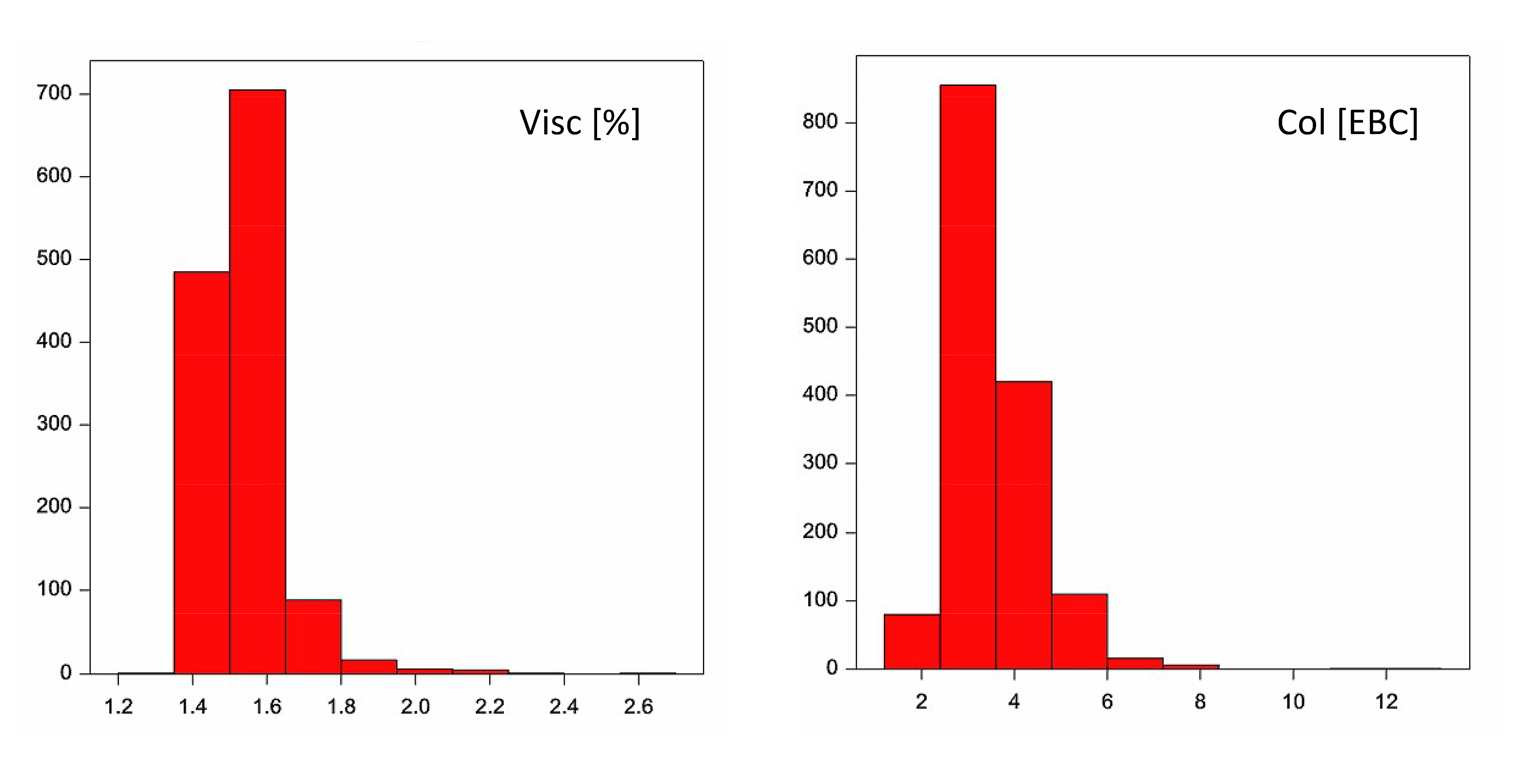

Supplement: Figure S1 — Histograms of the phenotypic trait distribution among cultivars. GY = grain yield, MY = marketable yield, TGW = thousand grain weight, HLW = hectoliter weight, KF = kernel formation, GF = glume fineness, SF = sieve fraction, K_RP = raw kernel protein content, M_RP = raw malt protein content, solN = soluble nitrogen, solP = soluble protein, Visc = viscosity, Col = color, Fria = friability, VZ45 = saccharification number VZ45°C, Extr = malt extract, FiAt = final attenuation, MQI = malting quality index. (ZIP) [file pone.0110046.s001.zip › Suppl_Fig_1-7_LZW_600dpi_.tif]

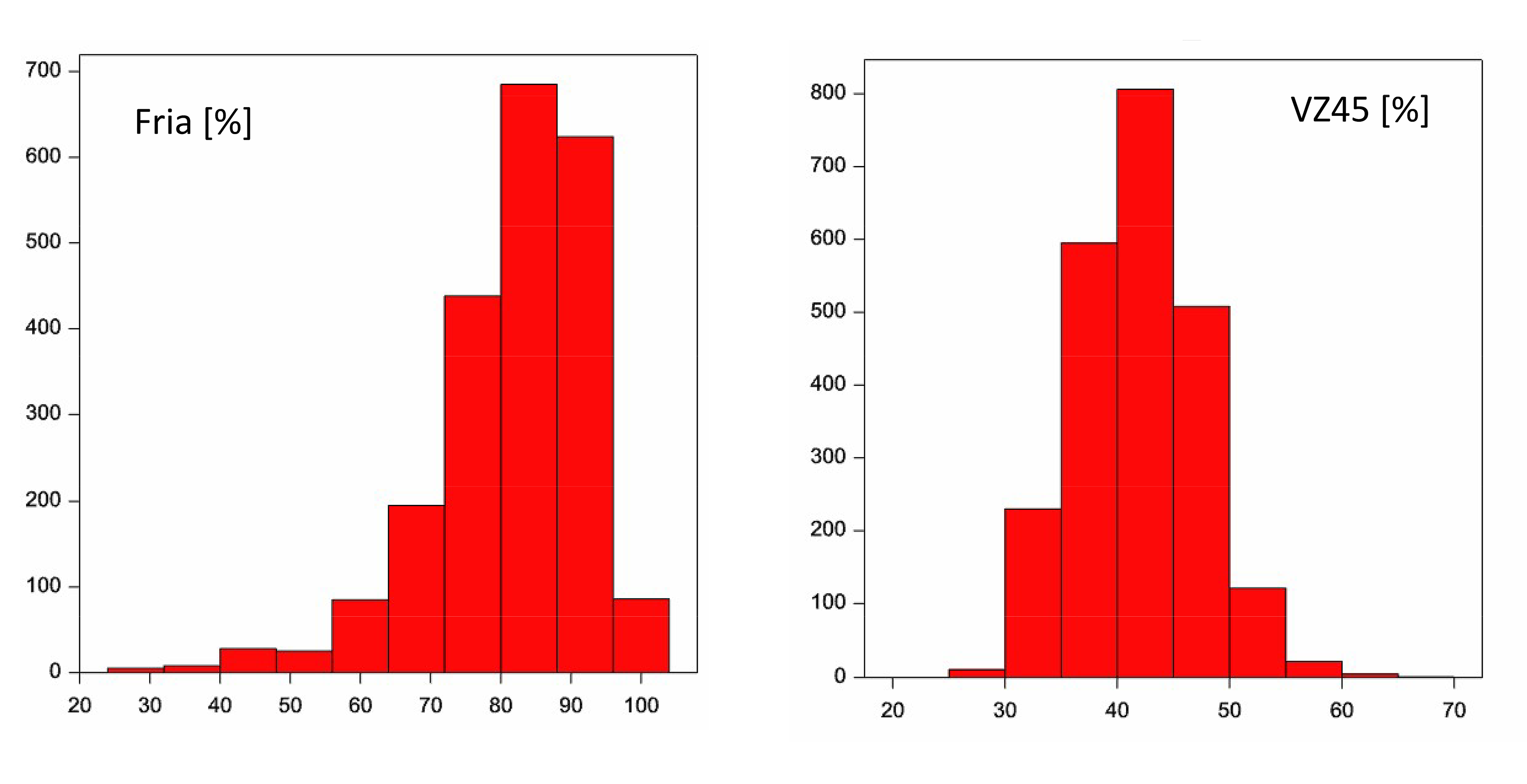

Supplement: Figure S1 — Histograms of the phenotypic trait distribution among cultivars. GY = grain yield, MY = marketable yield, TGW = thousand grain weight, HLW = hectoliter weight, KF = kernel formation, GF = glume fineness, SF = sieve fraction, K_RP = raw kernel protein content, M_RP = raw malt protein content, solN = soluble nitrogen, solP = soluble protein, Visc = viscosity, Col = color, Fria = friability, VZ45 = saccharification number VZ45°C, Extr = malt extract, FiAt = final attenuation, MQI = malting quality index. (ZIP) [file pone.0110046.s001.zip › Suppl_Fig_1-8_LZW_600dpi_.tif]

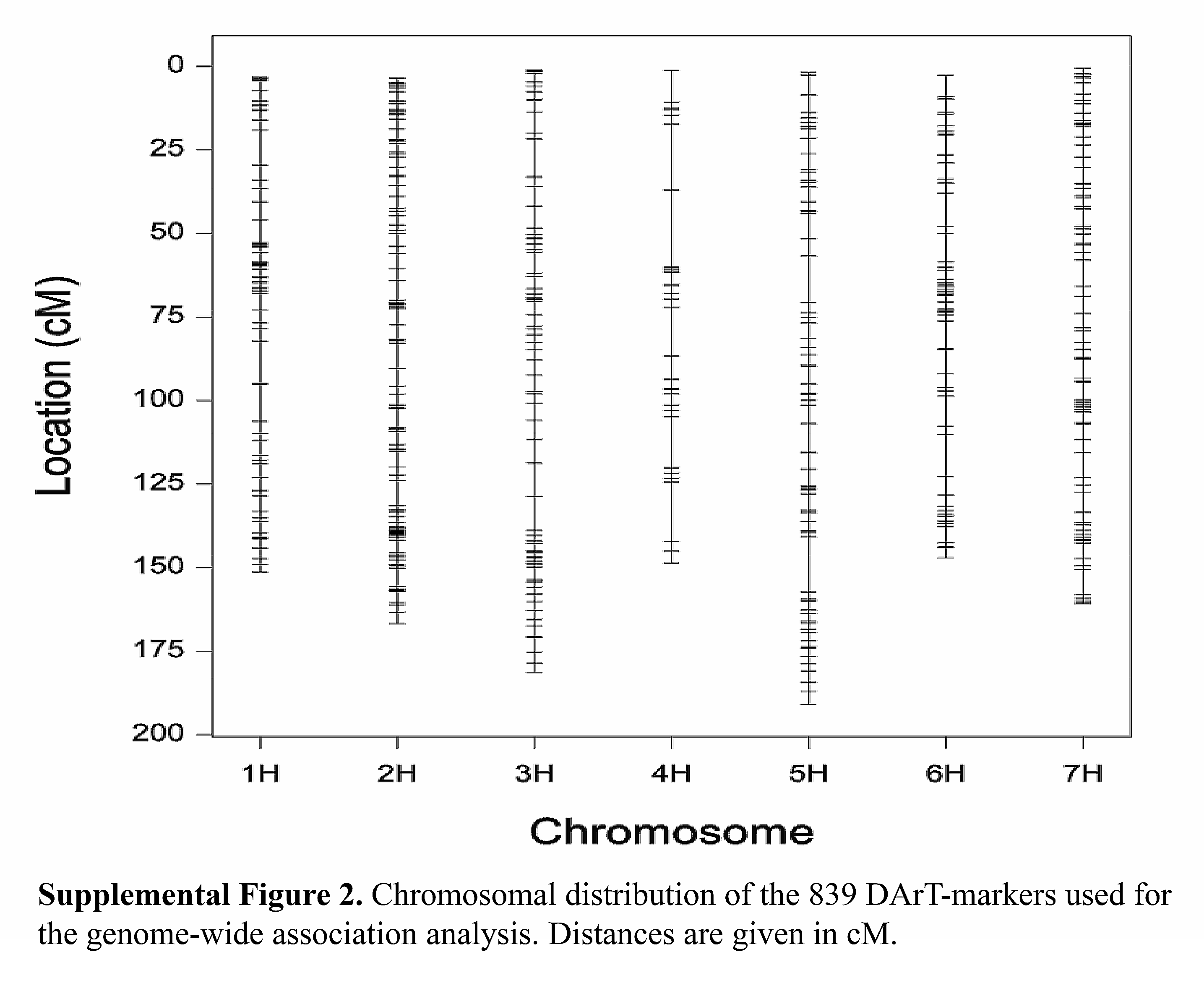

Supplement: Figure S2 — Chromosomal distribution of the 839 DArT markerDArT markers used for the genome-wide association analysis. Distances are given in cM. (TIF) [file pone.0110046.s002.tif]

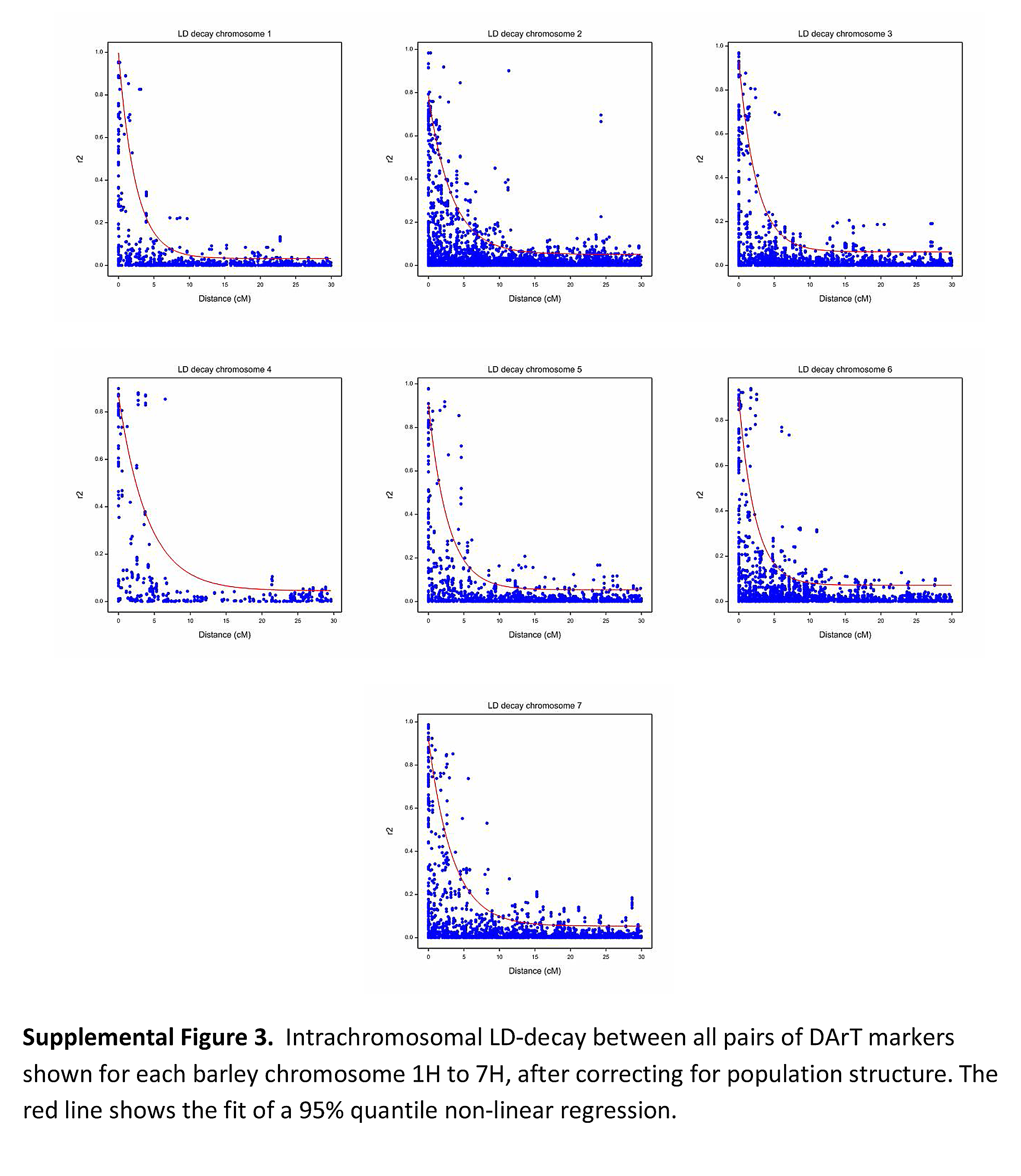

Supplement: Figure S3 — Intrachromosomal LD-decay between all pairs of DArT markers shown for each barley chromosome, 1H to 7H, after correcting for population structure. (TIF) [file pone.0110046.s003.tif]

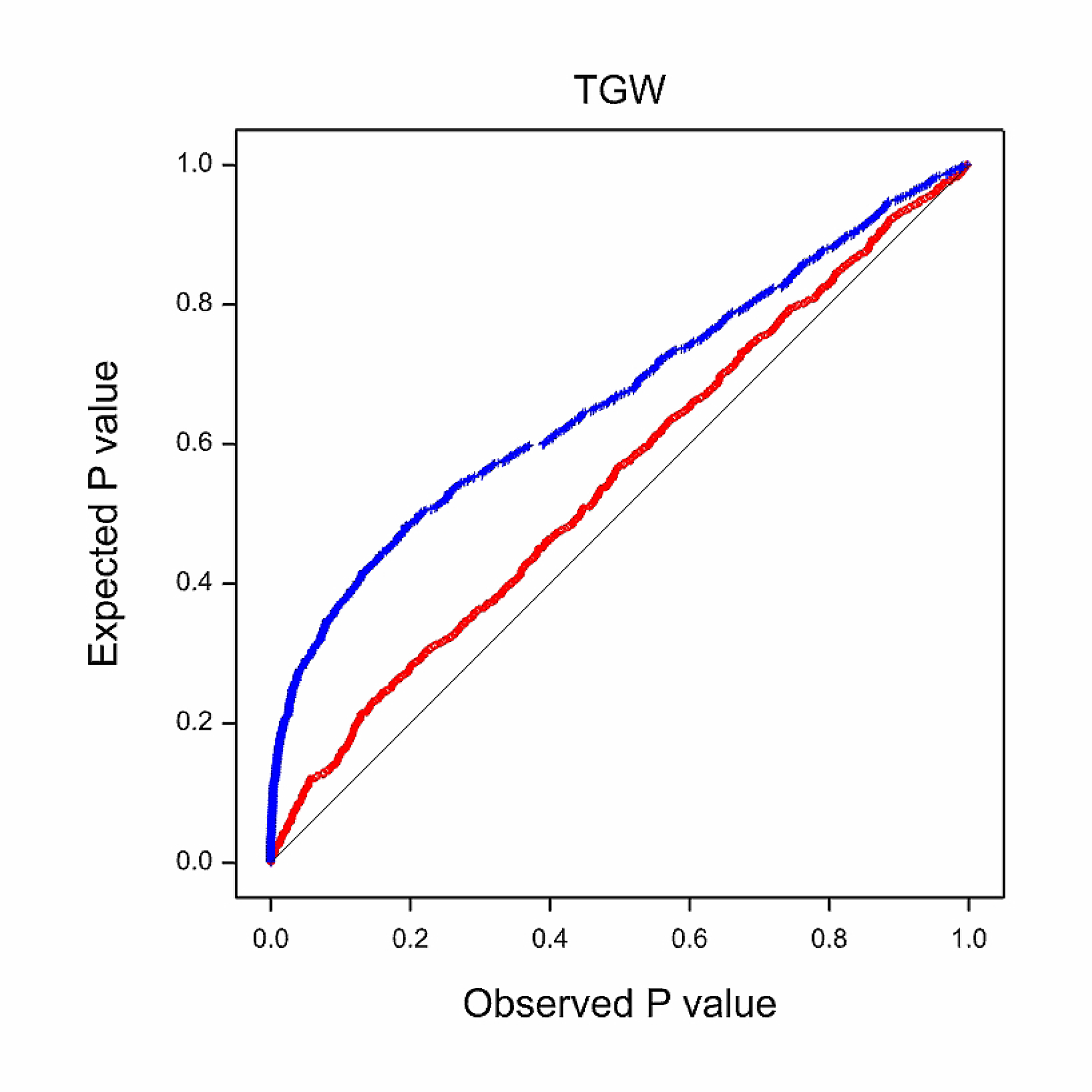

Supplement: Figure S4 — Comparison of P-values obtained by applying the naïve model (blue line) and the mixed linear model (MLM). The MLM incorporates corrections for population structure and kinship, based on PCA scores (red line). The comparison permits a check of the quality of the association results depicted for four traits (a) Grain yield (GY), (b) hectoliter weight (HLW), (c) kernel formation (KF), and (d) thousand grain weight (TGW). (ZIP) [file pone.0110046.s004.zip › Suppl_Fig_4-4_LZW_600dpi_.tif]

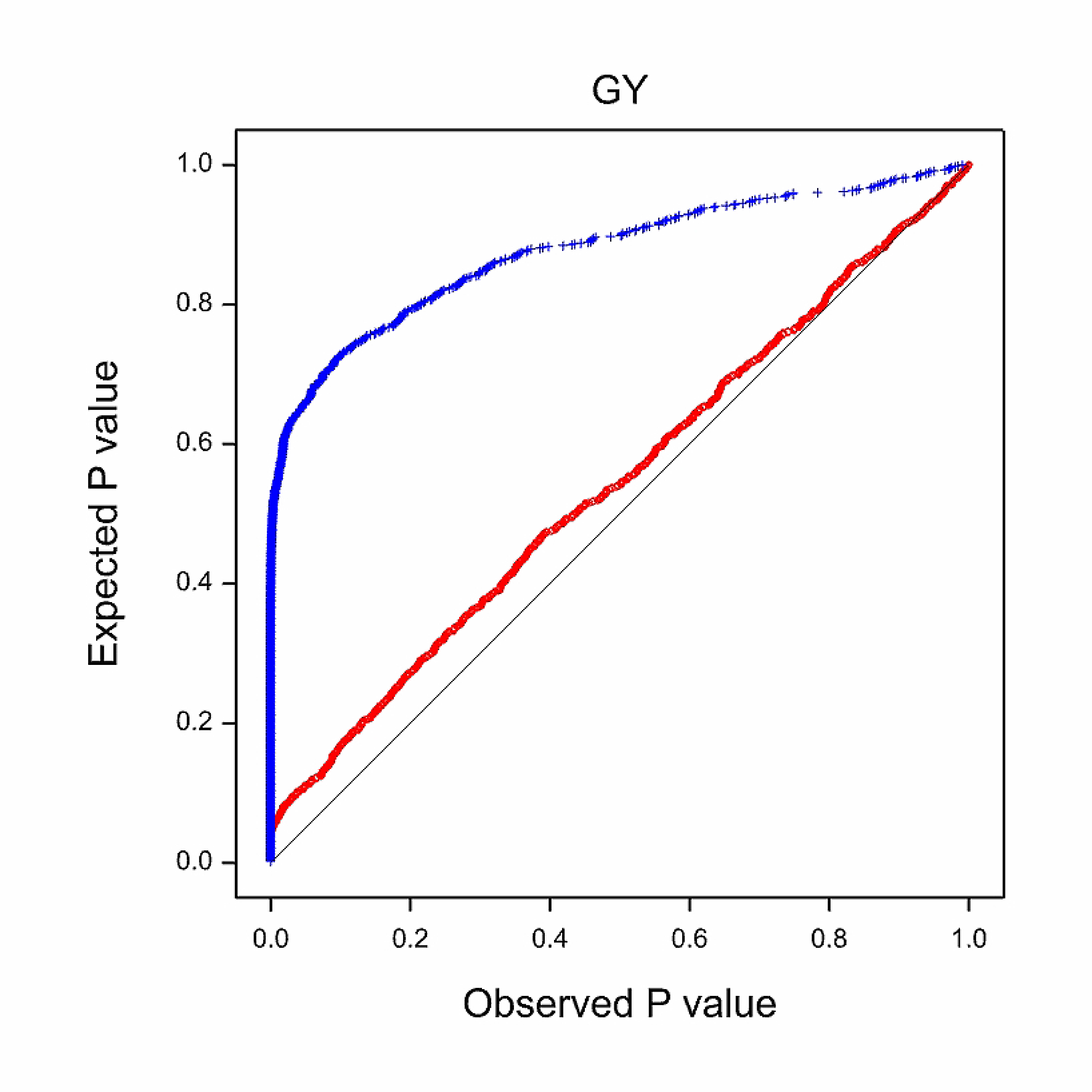

Supplement: Figure S4 — Comparison of P-values obtained by applying the naïve model (blue line) and the mixed linear model (MLM). The MLM incorporates corrections for population structure and kinship, based on PCA scores (red line). The comparison permits a check of the quality of the association results depicted for four traits (a) Grain yield (GY), (b) hectoliter weight (HLW), (c) kernel formation (KF), and (d) thousand grain weight (TGW). (ZIP) [file pone.0110046.s004.zip › Suppl_Fig_4-1_LZW_600dpi.tif]

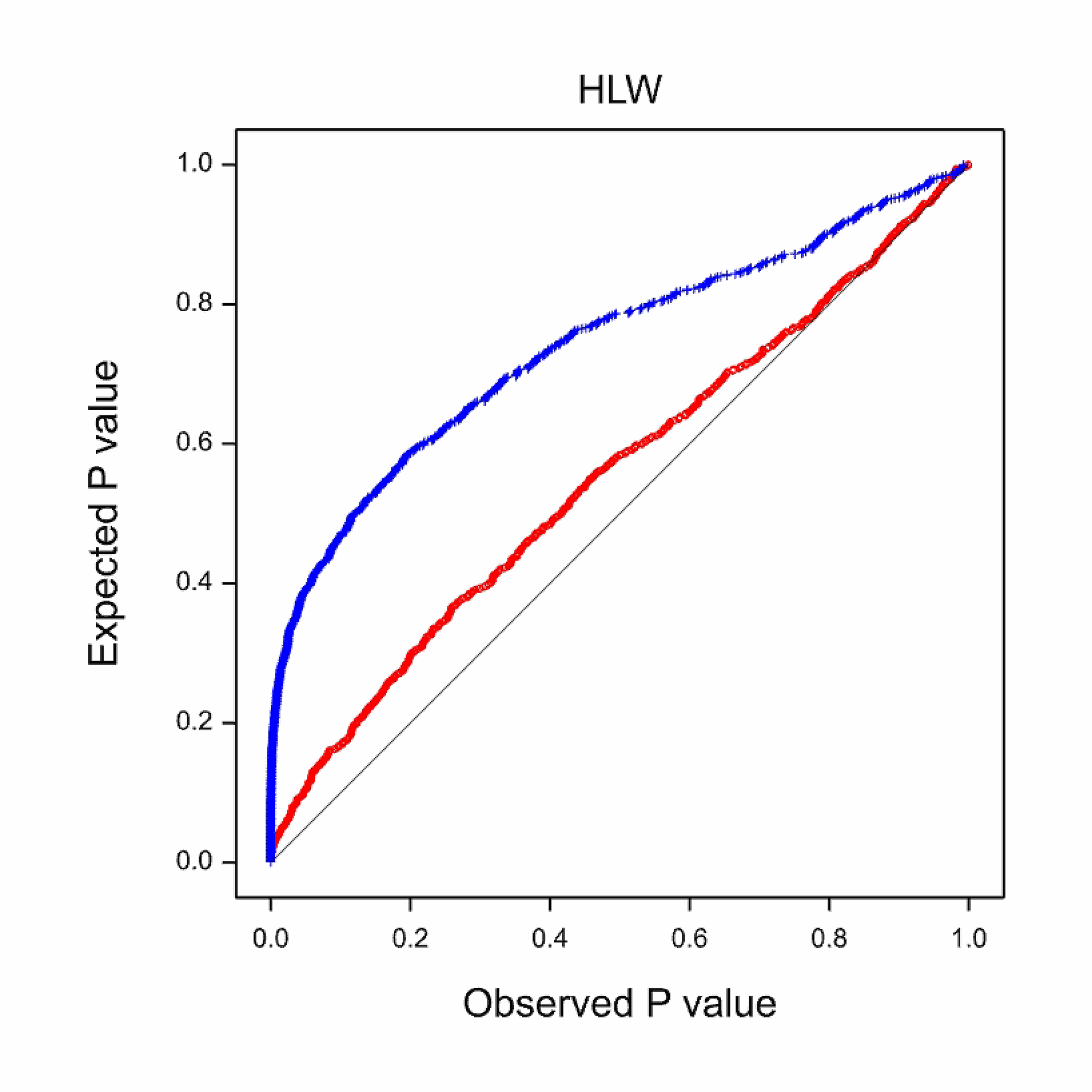

Supplement: Figure S4 — Comparison of P-values obtained by applying the naïve model (blue line) and the mixed linear model (MLM). The MLM incorporates corrections for population structure and kinship, based on PCA scores (red line). The comparison permits a check of the quality of the association results depicted for four traits (a) Grain yield (GY), (b) hectoliter weight (HLW), (c) kernel formation (KF), and (d) thousand grain weight (TGW). (ZIP) [file pone.0110046.s004.zip › Suppl_Fig_4-2_LZW_600dpi_.tif]

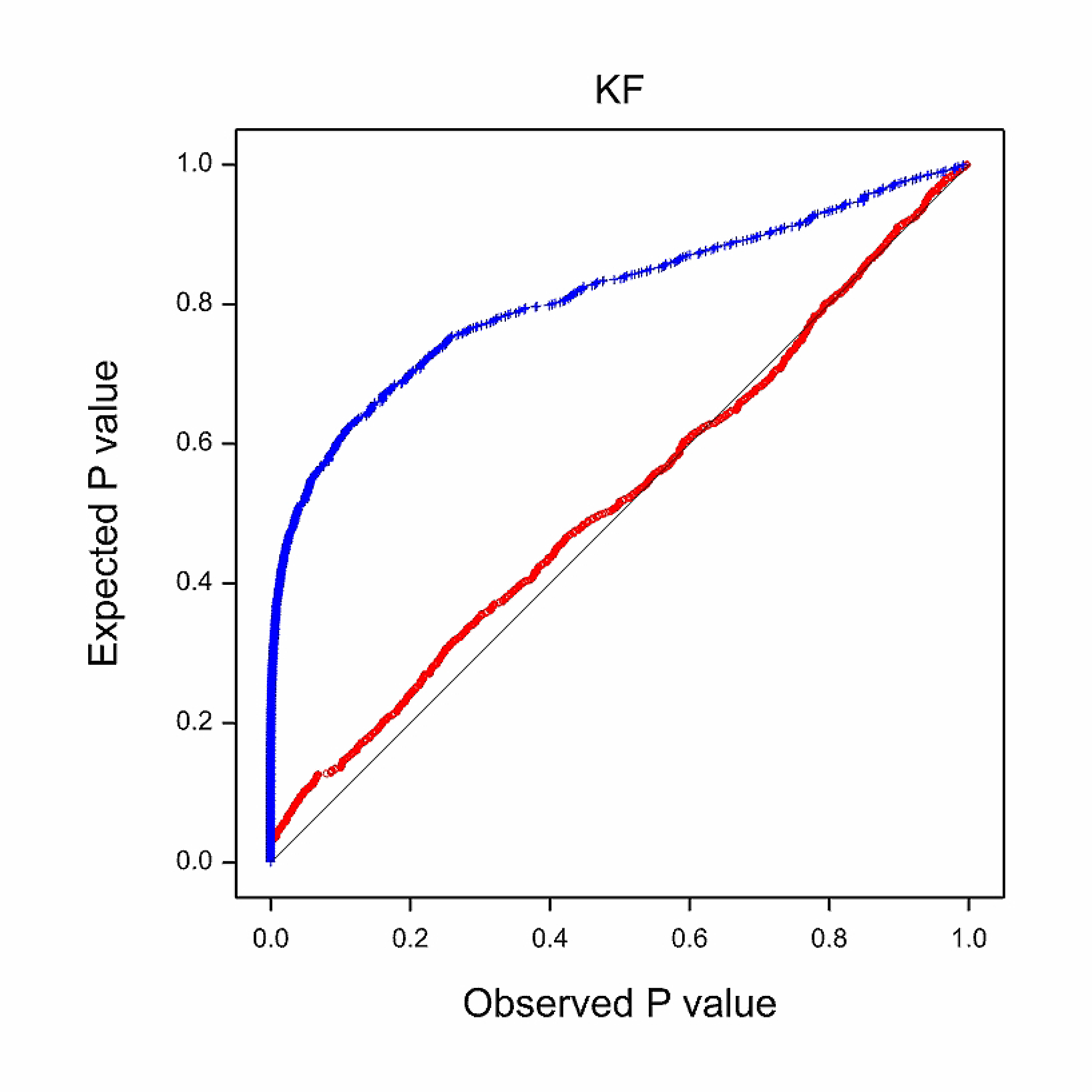

Supplement: Figure S4 — Comparison of P-values obtained by applying the naïve model (blue line) and the mixed linear model (MLM). The MLM incorporates corrections for population structure and kinship, based on PCA scores (red line). The comparison permits a check of the quality of the association results depicted for four traits (a) Grain yield (GY), (b) hectoliter weight (HLW), (c) kernel formation (KF), and (d) thousand grain weight (TGW). (ZIP) [file pone.0110046.s004.zip › Suppl_Fig_4-3_LZW_600dpi_.tif]

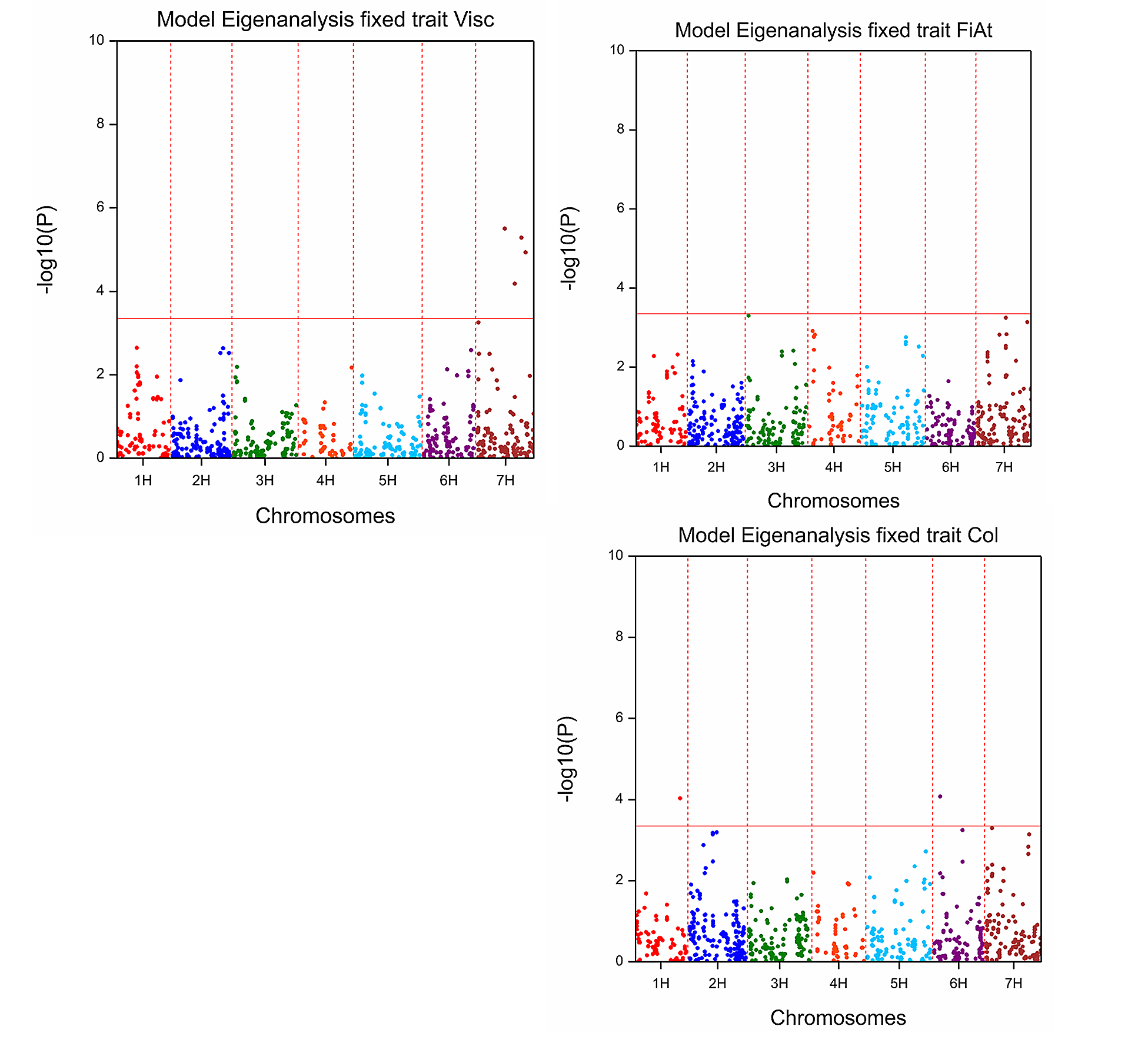

Supplement: Figure S5 — Manhattan plots show GWAS results from the PCA-corrected model for all kernel and malting parameters. GY = grain yield, MY = marketable yield, TGW = thousand grain weight, HLW = hectoliter weight, KF = kernel formation, GF = glume fineness, SF = sieve fraction, K_RP = raw kernel protein content, M_RP = raw malt protein content, solN = soluble nitrogen, solP = soluble protein, Visc = viscosity, Col = color, Fria = friability, VZ45 = saccharification number VZ45°C, Extr = malt extract, FiAt = final attenuation, MQI = malting quality index. (ZIP) [file pone.0110046.s005.zip › Suppl_Fig_5_Manhattan-5_LZW_600dpi_.tif]

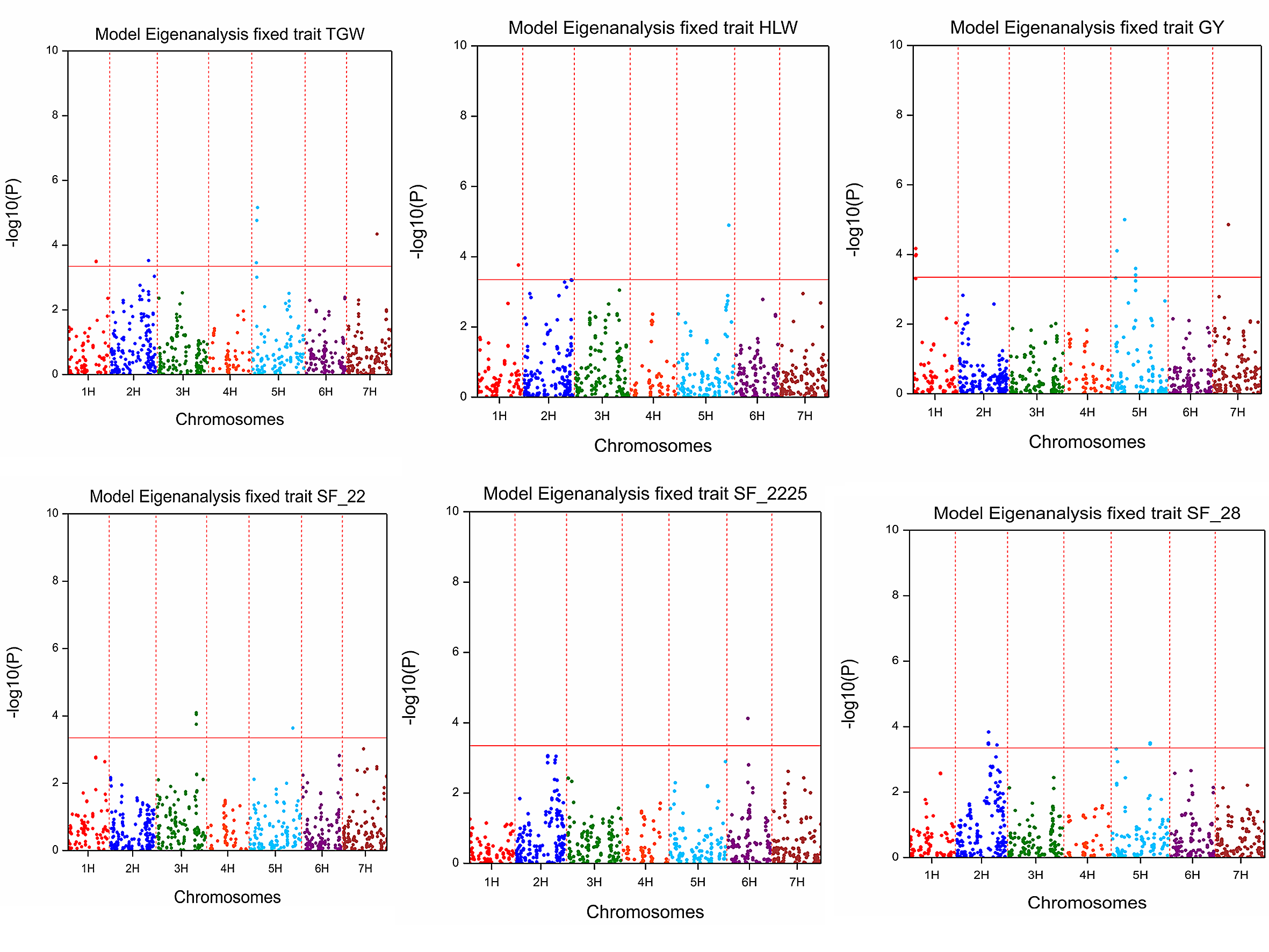

Supplement: Figure S5 — Manhattan plots show GWAS results from the PCA-corrected model for all kernel and malting parameters. GY = grain yield, MY = marketable yield, TGW = thousand grain weight, HLW = hectoliter weight, KF = kernel formation, GF = glume fineness, SF = sieve fraction, K_RP = raw kernel protein content, M_RP = raw malt protein content, solN = soluble nitrogen, solP = soluble protein, Visc = viscosity, Col = color, Fria = friability, VZ45 = saccharification number VZ45°C, Extr = malt extract, FiAt = final attenuation, MQI = malting quality index. (ZIP) [file pone.0110046.s005.zip › Suppl_Fig_5_Manhattan-1_LZW_600dpi_.tif]

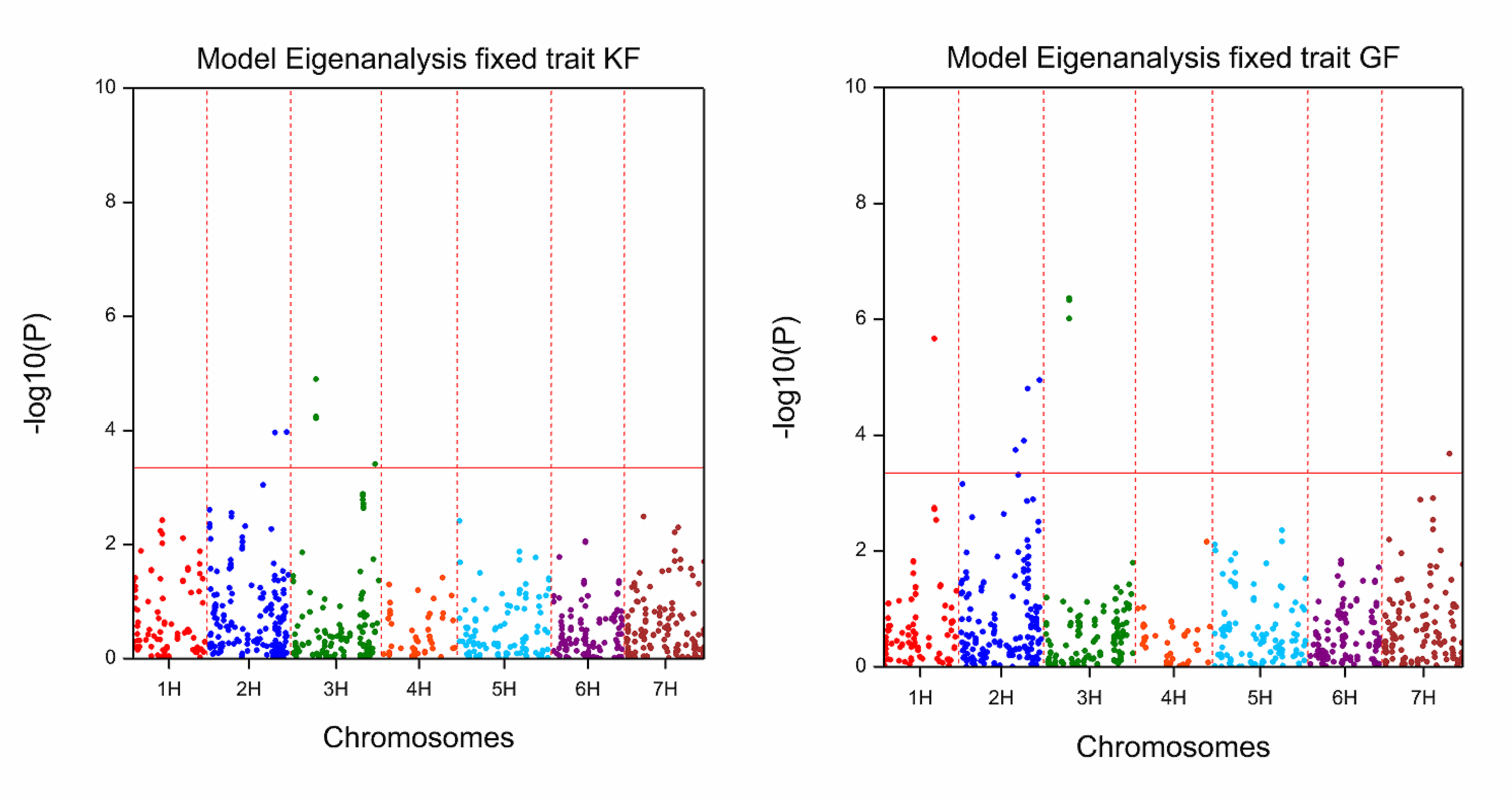

Supplement: Figure S5 — Manhattan plots show GWAS results from the PCA-corrected model for all kernel and malting parameters. GY = grain yield, MY = marketable yield, TGW = thousand grain weight, HLW = hectoliter weight, KF = kernel formation, GF = glume fineness, SF = sieve fraction, K_RP = raw kernel protein content, M_RP = raw malt protein content, solN = soluble nitrogen, solP = soluble protein, Visc = viscosity, Col = color, Fria = friability, VZ45 = saccharification number VZ45°C, Extr = malt extract, FiAt = final attenuation, MQI = malting quality index. (ZIP) [file pone.0110046.s005.zip › Suppl_Fig_5_Manhattan-2_LZW_600dpi_.tif]

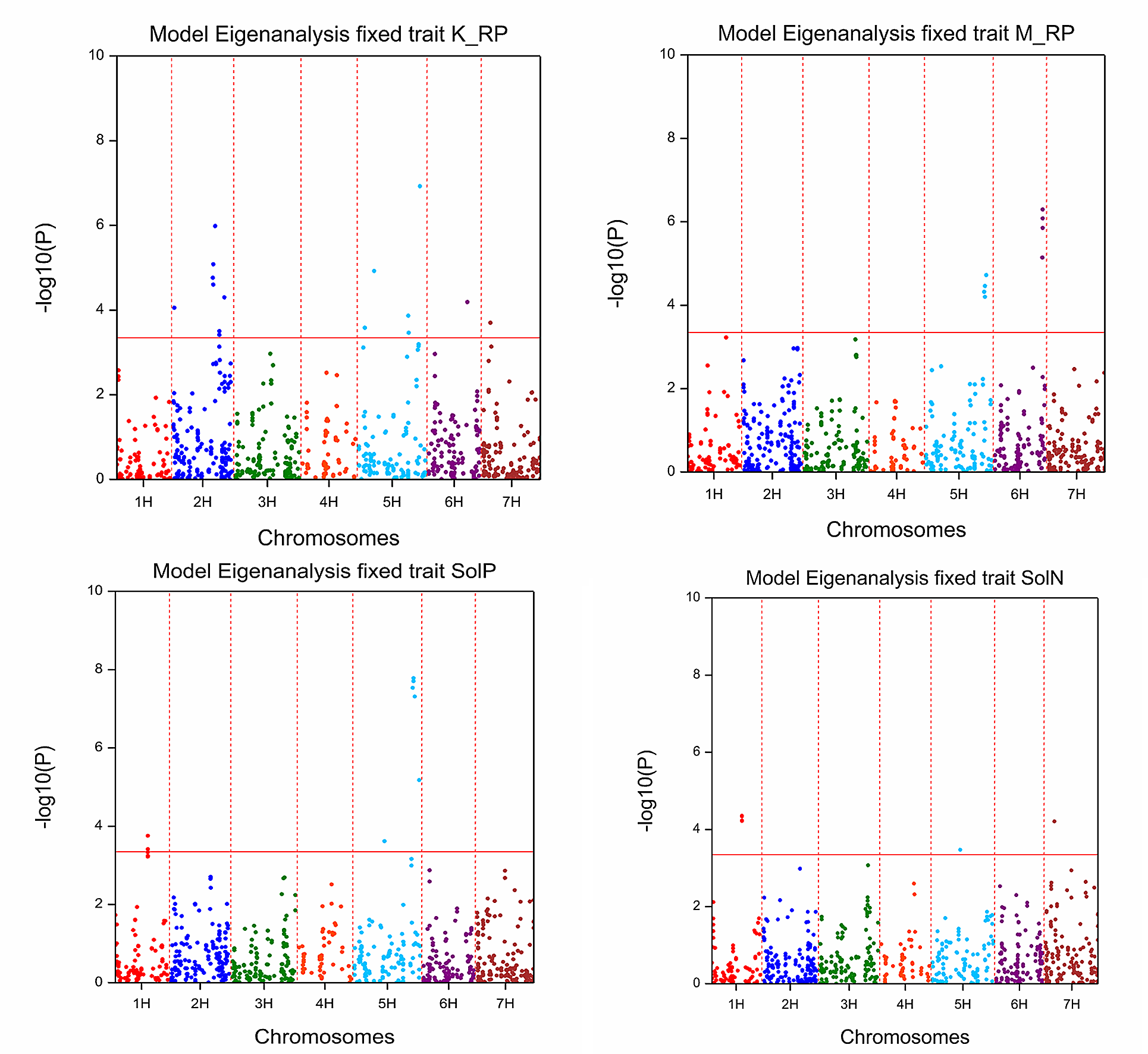

Supplement: Figure S5 — Manhattan plots show GWAS results from the PCA-corrected model for all kernel and malting parameters. GY = grain yield, MY = marketable yield, TGW = thousand grain weight, HLW = hectoliter weight, KF = kernel formation, GF = glume fineness, SF = sieve fraction, K_RP = raw kernel protein content, M_RP = raw malt protein content, solN = soluble nitrogen, solP = soluble protein, Visc = viscosity, Col = color, Fria = friability, VZ45 = saccharification number VZ45°C, Extr = malt extract, FiAt = final attenuation, MQI = malting quality index. (ZIP) [file pone.0110046.s005.zip › Suppl_Fig_5_Manhattan-3_LZW_600dpi_.tif]

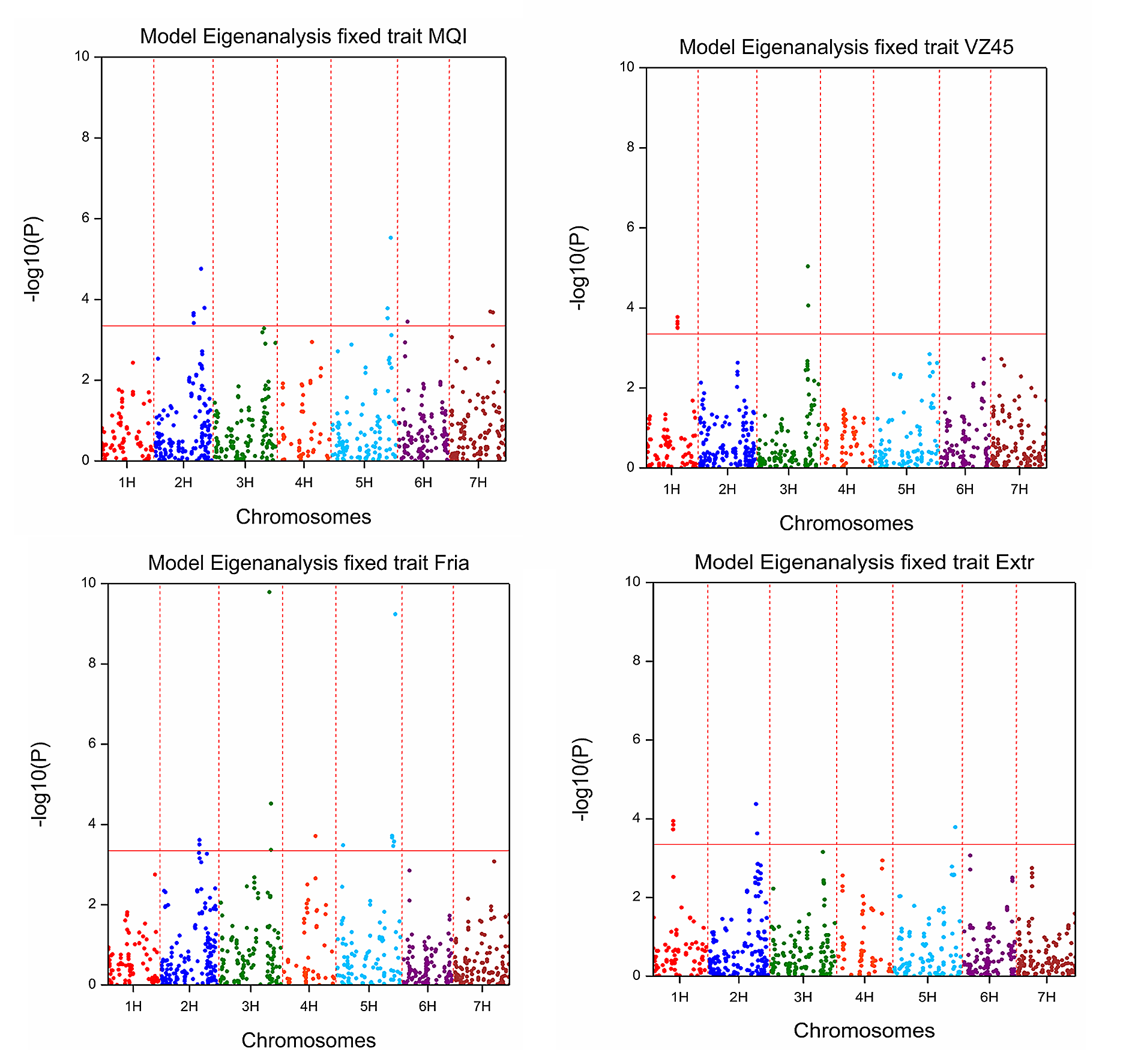

Supplement: Figure S5 — Manhattan plots show GWAS results from the PCA-corrected model for all kernel and malting parameters. GY = grain yield, MY = marketable yield, TGW = thousand grain weight, HLW = hectoliter weight, KF = kernel formation, GF = glume fineness, SF = sieve fraction, K_RP = raw kernel protein content, M_RP = raw malt protein content, solN = soluble nitrogen, solP = soluble protein, Visc = viscosity, Col = color, Fria = friability, VZ45 = saccharification number VZ45°C, Extr = malt extract, FiAt = final attenuation, MQI = malting quality index. (ZIP) [file pone.0110046.s005.zip › Suppl_Fig_5_Manhattan-4_LZW_600dpi_.tif]
